# Efficacy and tolerability of FDA-approved atypical antipsychotics for the treatment of bipolar depression: a systematic review and network meta-analysis

**DOI:** 10.1192/j.eurpsy.2024.25

**Published:** 2024-03-15

**Authors:** Shaoli Li, Chenyue Xu, Shaohua Hu, Jianbo Lai

**Affiliations:** 1 Department of Psychiatry, the First Affiliated Hospital, Zhejiang University School of Medicine, Hangzhou 310003, China; 2 Department of Medical Oncology, the Second Affiliated Hospital, Zhejiang University School of Medicine, Hangzhou 310009, China; 3 The Key Laboratory of Mental Disorders’ Management, Zhejiang Province, Hangzhou 310003, China; 4 Brain Research Institute, Zhejiang University, Hangzhou 310058, China; 5 Zhejiang Engineering Center for Mathematical Mental Health, Hangzhou 310003, China; 6 The MOE Frontier Science Center for Brain Science & Brain-machine Integration, Zhejiang University, Hangzhou 310058, China

**Keywords:** atypical antipsychotic, bipolar disorder, efficacy, network meta-analysis, tolerability

## Abstract

We employed a Bayesian network meta-analysis for comparison of the efficacy and tolerability of US Food and Drug Administration (FDA)-approved atypical antipsychotics (AAPs) for the treatment of bipolar patients with depressive episodes. Sixteen randomized controlled trials with 7234 patients treated by one of the five AAPs (cariprazine, lumateperone, lurasidone, olanzapine, and quetiapine) were included. For the response rate (defined as an improvement of ≥50% from baseline on the Montgomery-Åsberg Depression Rating Scale [MADRS]), all AAPs were more efficacious than placebo. For the remission rate (defined as the endpoint of MADRS ≤12 or ≤ 10), cariprazine, lurasidone, olanzapine, and quetiapine had higher remission rates than placebo. In terms of tolerability, olanzapine was unexpectedly associated with lower odds of all-cause discontinuation in comparison with placebo, whereas quetiapine was associated with higher odds of discontinuation due to adverse events than placebo. Compared with placebo, lumateperone, olanzapine, and quetiapine showed higher odds of somnolence. Lumateperone had a lower rate of ≥ weight gain of 7% than placebo and other treatments. Olanzapine was associated with a significant increase from baseline in total cholesterol and triglycerides than placebo. These findings inform individualized prescriptions of AAPs for treating bipolar depression in clinical practice.

## Introduction

Bipolar disorder (BD) manifests as a highly recurrent mood disorder that affects more than 1% population worldwide [1, 2]. Compared to manic or hypomanic phases, depressive phases are more commonly presented and can last longer [3]. Currently, bipolar depression remains a major clinical challenge regarding its complex trajectory of relapse, remission, recurrence, and treatment response [2, 4].

Despite the emergence of various non-pharmacological treatment options for bipolar depression (e.g., lifestyle changes, physical therapy, and psychotherapy) [5], pharmacological treatment including atypical antipsychotics (AAPs), anticonvulsants, and lithium salts remains the cornerstone for most individuals with BD [6]. In the Royal Australian and New Zealand College of Psychiatrists Clinical Guidelines for Mood Disorders 2020, two broad groups of medications, mood stabilizing agents (lithium, lamotrigine, and valproate) and AAPs (quetiapine, cariprazine, and lurasidone), are recommended for treating depressive episodes of BD, and mood stabilizing agents are considered to be more preferable to AAPs [7]. In recent decades, however, there has been a trend that more AAPs and fewer mood stabilizers have been prescribed in BD treatment [8]. Notably, only a few AAPs, but no mood-stabilizing agent, have been approved by the US Food and Drug Administration (FDA) for the treatment of acute bipolar depression.

Since the approval of quetiapine for bipolar depression in 2004, other AAPs including olanzapine-fluoxetine combination (2012), lurasidone (2013), cariprazine (2019), and lumateperone (2021) have intermittently gained approval from FDA. Nonetheless, the treatment outcome of antipsychotics for patients with bipolar depression varied across different studies, with a treatment response rate ranging from 39.0 to 69.1%, whereas the remission rate varied from 26.0 to 70.1% [9–12]. Each agent owns its pros and cons in the clinical application regarding their different pharmacokinetic and pharmacodynamic characteristics (see eTable 1). In the 2018 guidelines from the Canadian Network for Mood and Anxiety Treatments (CANMAT) and the International Society for Bipolar Disorders (ISBD), only quetiapine or lurasidone monotherapy, as well as lurasidone in combination with valproate or lithium, are recommended as the first-line treatment for the acute phase of bipolar depression, whereas cariprazine, and olanzapine-fluoxetine combination are listed as second-line choices [13]. In a recent network meta-analysis (NMA) regarding AAPs for bipolar depression [9], lurasidone, quetiapine, olanzapine, and cariprazine all showed better treatment response than placebo assessed by the change in score on the Montgomery-Åsberg Depression Rating Scale (MADRS) scores. Lurasidone showed similar odds of response to olanzapine and quetiapine but was superior to cariprazine [9]. Compared to placebo, lurasidone had a similar effect on weight change, whereas olanzapine, quetiapine, and cariprazine had a greater weight gain [9].

As a newly approved agent, lumateperone has not yet been mentioned in any international guidelines for bipolar treatment or involved in previous meta-analysis or NMA studies. In this study, we aimed to conduct an immediate NMA update to deepen our understanding regarding the five FDA-approved AAPs for treating bipolar depression, specifically in terms of response rate and all-cause discontinuation. Additionally, our study also explored secondary outcomes such as remission rate, adverse events, and metabolic outcomes. We hypothesized that the efficacy and tolerability of the aforementioned five AAPs (cariprazine, lumateperone, lurasidone, olanzapine, and quetiapine) would be comparable in the treatment of bipolar depression.

## Methods

This NMA has been registered in the PROSPERO (Registration ID: CRD42023390502) and strictly followed guidelines for the Preferred Reporting Items for Systematic Reviews and Meta-analyses (PRISMA) [14]. The steps of literature retrieval and inclusion, data extraction and collation, as well as quality control, were independently performed by two researchers (S.L. and C.X.).

### Search strategy and study selection

A complete literature review of randomized controlled trials (RCTs) on the FDA-approved AAPs for bipolar depression (cariprazine, lumateperone, lurasidone, olanzapine, and quetiapine) was performed based on a combination of free-text terms and controlled vocabulary when needed. The most recent NMA for bipolar depression was for studies that had completion dates before May 2020 [9]. This update also included a search of PubMed, Embase, and the Cochrane Library for trials published between May 2020 and 3^rd^ August 2022. The references of relevant systematic reviews or meta-analyses were screened to track additional studies. The inclusion and exclusion criteria were updated from the previous NMA, and we included double-blinded RCTs comparing the FDA-approved AAPs with a placebo or another FDA-approved AAP as monotherapy for treating adults (aged ≥18), with a primary diagnosis of BD (at least 50% of participants with bipolar I disorder (BPAD1)), and documented at least one outcome of interest at study endpoint (see Table 1). The detailed search strategy can be found in Supplementary materials.

**Table 1.** The inclusion and exclusion criteria in this study

Criterion	Inclusion	Exclusion
Patient population	Adults with bipolar depression (>18 year–old); ≥ 50% of the subjects met the diagnosis of bipolar I disorder.	<50% subjects with bipolar I disorder; <18-year-old.
Interventions	Monotherapy with US FDA–approved AAP: Cariprazine; Olanzapine; Quetiapine; Lurasidone; Lumateperone.	Any treatment other than those listed in the inclusion criteria; Any treatment listed in the inclusion criteria but used as an adjunctive.
Comparisons	Any of the aforementioned five medications or placebo	Comparators are not listed in the inclusion criteria.
outcomes	Studies reporting at least one of the following outcomes: Response (≥ 50% improvement in MADRS from baseline); Remission (MADRS score ≤ 12 and ≤ 10 at the endpoint); All–cause discontinuation; Discontinuation due to adverse events; Somnolence; Headache; Nausea; ≥ 7% weight gain; Change in total cholesterol from baseline; Change in triglycerides from baseline; Change in total glucose level from baseline.	Studies not report any of the outcomes included in the inclusion criteria.
Study design	RCTs	Non-RCTs; Observational study; Case study; Pharmacology study.

Abbreviations: AAP, atypical antipsychotic; FDA, Food and Drug Administration; MADRS, Montgomery–Åsberg Depression Rating Scale; RCTs, randomized controlled trials.

### Primary and secondary outcomes variables

The primary outcome included two parameters, endpoint response rate (defined as ≥50% improvement in MADRS compared to baseline) and acceptability (measured by treatment all-cause discontinuation). All-cause discontinuation was adopted as an indicator reflecting the treatment acceptability as it encompassed both efficacy and tolerability. The Secondary outcomes included remission rate (defined as an endpoint MADRS score ≤ 12 or ≤ 10), discontinuation due to adverse events, adverse events (rate of somnolence, headache, and nausea), and metabolic outcomes (rate of ≥7% weight gain, change in serum levels of total cholesterol, triglycerides, and blood glucose) reported at the study endpoint.

### Data extraction

Two researchers (S.L. and C.X.) independently reviewed the full text of all eligible studies. Any discrepancy was resolved by discussion, and if disagreement remained, a final decision was made by the senior author (J.L.). Data were extracted on outcomes variables, general characteristics (including the first author, publication year, total sample size, and follow-up), and patient characteristics (including age, gender, weight, type of BD, treatment, duration, and baseline MADRS score). For all analyses, outcomes were recorded as close to 8 weeks as possible. If data at 8 weeks was absent, an alternative timepoint closest to 8 weeks (ranging from 4 to 12 weeks, and the longer duration was preferred if equidistant) was preferred. Missing standard deviations (SDs) for continuous outcomes were calculated by standard errors (SEs), 95% confidence intervals, and *P-*value or SDs of baseline and endpoint values. All data were obtained within the published studies. No additional data was requested by contacting the authors.

### Statistical analysis

Stata software (version 14.0, Stata Corp, TX, USA) and WinBUGS (version 1.4.3, MRC Biostatistics Unit, Cambridge, UK) were used to perform all the analyses. As for results, continuous variables were presented as standardized mean differences (SMD, Cohen’s d), and discontinuous variables with odds ratios (ORs) and their 95% credibility interval (95% Crl). For categorical data, a correction of 0.5 zero-cell was applied during the meta-analysis procedure.

For each outcome mentioned above, an initial meta-analysis was performed for direct pairwise comparison with fixed or random effects, which was followed by a Bayesian random-effects NMA to simultaneously compare all AAPs using the Markov-chain Monte Carlo method in compliance with the assumption of transitivity. Four chains were run, generating 200,000 iterations and discarding the first 20,000 burn-ins. The convergence of models was evaluated by trace plots and Brooks-Gelman-Rubin statistics. The model fit was assessed by comparing the totresdev and the data point of the study. The surface under the cumulative ranking curve (SUCRA) was calculated to rank the AAPs for each outcome [15]. All *P* values are two-sided, with *P*<0.05 considered statistically significant.

### Quality of evidence and heterogeneity

Two authors (S.L. and C.X.) independently evaluated the quality of the included studies with the Cochrane Collaboration tool for assessing the bias risk in RCTs [16]. Disagreements were discussed and resolved through consensus.

Transitivity was defined based on the assumption that the distribution of effect modifiers across different studies was sufficiently similar so that indirect comparisons could be validly used to compare two AAP alternatives. In the current NMA, we assessed this assumption by comparing the distribution of clinical and methodological variables which may serve as effect modifiers among AAP comparisons. The heterogeneity among the included studies was evaluated by I^2^ in the pairwise meta-analysis and Tau [2] in the NMA. The confidence of evidence was assessed using the Grading of Recommendation, Assessment, Development, and Evaluation (GRADE) method for NMA [17, 18]. Since no closed loop was in the current NMA, an assessment of the inconsistency was waived in this study.

### Sensitivity analysis and publication bias

We conducted a sensitivity analysis to assess the quality and consistency of the results by individually excluding each study. Additionally, publication bias was evaluated using Egger’s test and a visual inspection of asymmetry in the funnel plot.

## Results

### Literature review

**Figure 1.**
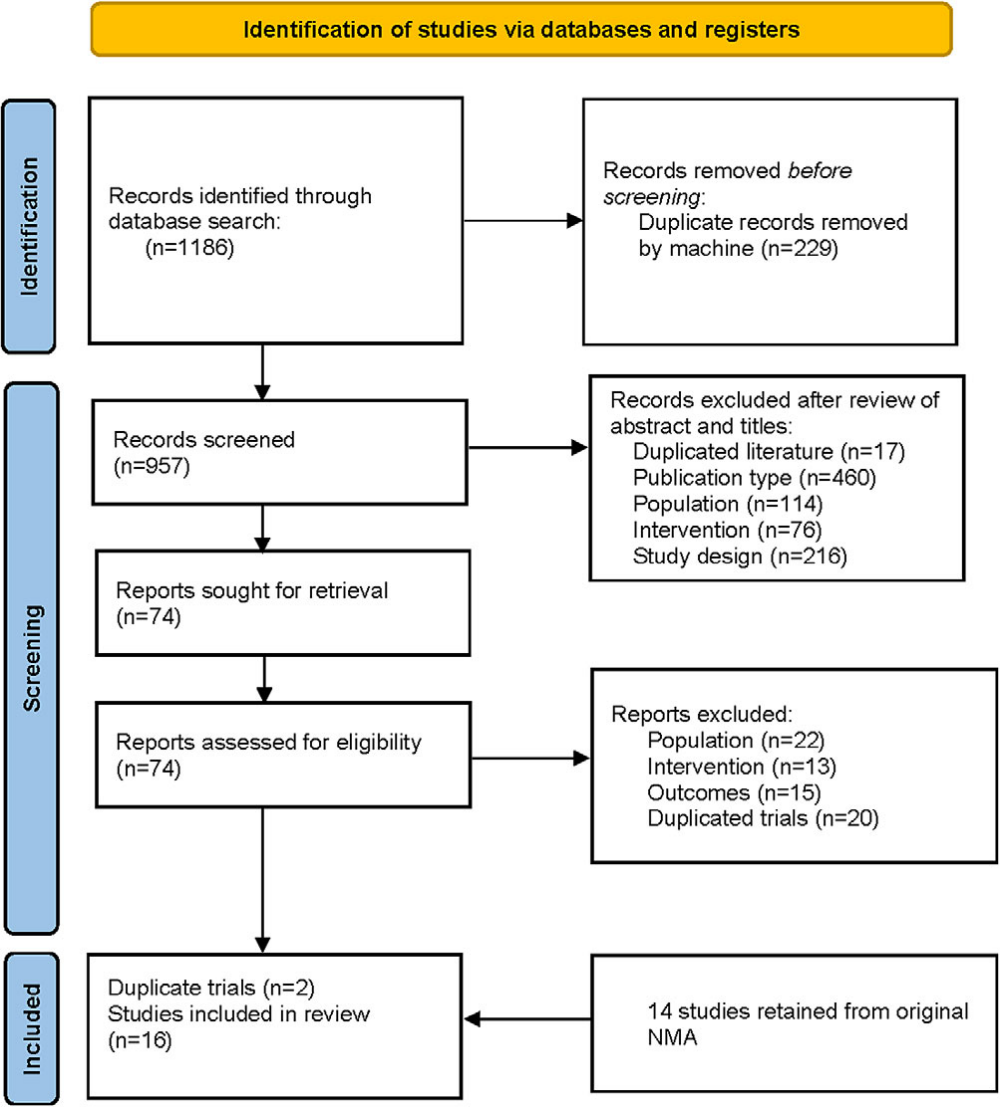
Flow diagram of literature search.

This updated systematic literature review screened 1,186 records in PubMed, Embase, and the Cochrane Library. Seventy-four full-text articles were examined, with only 4 meeting the criteria for inclusion, and another 14 trials were obtained from the previous NMA [9]. After excluding the duplicated records, a total of 16 trials were included in the final analysis (see Figure 1). Overall, this NMA included 2500 participants in the placebo group and 4734 treated with one of the following AAPs: cariprazine, lumateperone, lurasidone, olanzapine, and quetiapine.

### Study characteristics

All studies were double-blind and placebo-controlled RCTs carried out across multiple sites. Most trials were multi-national, only four trials recruited participants exclusively from sites in the United States [19–22], and two studies recruited participants exclusively from China [23, 24]. Most trials were carried out for 8 weeks in duration, whereas six studies lasted for only 6 weeks. General characteristics of eligible studies were shown in detail (see Table 2). The study subjects were comparable in terms of age (mean value, 29.2–45.0 years old), sex distribution (34.3–48.1% male), and MADRS score at baseline (mean value, 26.9–32.0). Average body weight at baseline was reported in 11 studies, ranging from 63.9 to 88.8 kg. Half of these studies solely enrolled patients with BPAD1, whereas the other half included both patients with BPAD1 and bipolar II disorder (BPAD2).

**Table 2.** Design and baseline characteristics of subjects in included studies

				Baseline characteristics				
Study (year)	n	Duration/week	Agent and dosage/mg	Age/years old	Male (%)	Weight/kilograms	Bipolar I (%)	MADRS score
Calabrese et al. (2005) [19]	511	8	Quetiapine IR 300 mg; Quetiapine IR 600 mg; Placebo	37.4	41.9%	NR	66.9%	30.4
Durgam et al. (2016) [29]	571	8	Cariprazine 0.75 mg; Cariprazine 1.5 mg; Cariprazine 3.0 mg; Placebo	41.9	37.7%	80.9	100.0%	30.6
Earley et al. (2018) [30]	480	6	Cariprazine 1.5 mg; Cariprazine 3.0 mg; Placebo	42.8	40.8%	86.5	100.0%	30.6
Earley et al. (2019) [31]	490	6	Cariprazine 1.5 mg; Cariprazine 3.0 mg; Placebo	43.6	37.3%	84.8	100%	31.4
Li et al. (2016) [23]	279	8	Quetiapine XR 300 mg; Placebo	33.1	48.1%	64.5	50.9%	28.7
Loebel et al. (2014) [32]	485	6	Lurasidone 20–60 mg; Lurasidone 80–120 mg; Placebo	41.5	41.4%	77.2	100%	30.5
McElroy et al. (2010) [33]	582	8	Quetiapine IR 300 mg; Quetiapine IR 600 mg; Placebo	38.5	36.9%	80.8	64.0%	26.9
Suppes et al. (2010) [20]	270	8	Quetiapine XR 300 mg; Placebo	39.5	35.5%	88.8	80.4%	30.0
Thase et al. (2006) [21]	467	8	Quetiapine IR 300 mg; Quetiapine IR 600 mg; Placebo	37.7	43.1%	NR	67.4%	30.2
Tohen et al. (2003) [25]	747	8	Olanzapine >5 mg (mean: 9.7 mg); Placebo	42.0	41.5%	NR	100.0%	32.0
Tohen et al. (2012) [34]	514	6	Olanzapine 10–20 mg; Placebo	35.5	44.3%	NR	100.0%	29.0
Wang et al. (2014) [24]	68	8	Olanzapine 10–20 mg; Placebo	29.2	41.2%	63.9	100.0%	28.6
Young et al. (2010) [35]	647	8	Quetiapine IR 300 mg; Quetiapine IR 600 mg; Placebo	42.2	41.6%	75.5	61.6%	28.3
Yatham et al. (2020) [22]	224	8	Cariprazine 0.25–0.75 mg; Cariprazine 1.5 mg–3.0 mg; Placebo	38.9	34.3%	NR	72.7%	30.4
Kato et al. (2020) [11]	522	6	Lurasidone 20–60 mg; Lurasidone 80–120 mg; Placebo	42.4	46.9%	72.4	100%	30.8
Calabrese et al. (2021) [10]	377	6	Lumateperone 60 mg; Placebo	45.0	41.9%	79.1	79.8%	30.5

Abbreviations: IR, immediate release; NR, not reported; XR, extended-release.

### Direct pairwise meta-analysis

The results of direct pairwise (cariprazine/lumateperone/lurasidone/olanzapine/quetiapine versus placebo) meta-analysis were shown in Supplementary material eFigures 1 and 2. For primary outcomes, the odds of response rate were significantly higher for cariprazine, lumateperone, lurasidone, olanzapine, and quetiapine compared with placebo. All-cause discontinuation rates for cariprazine, lumateperone, lurasidone, and quetiapine were comparable to that of the placebo. Nonetheless, the rate of all-cause discontinuation for olanzapine was significantly lower than that of the placebo.

### NMA results

#### Evidence network for the primary outcomes

**Figure 2.**
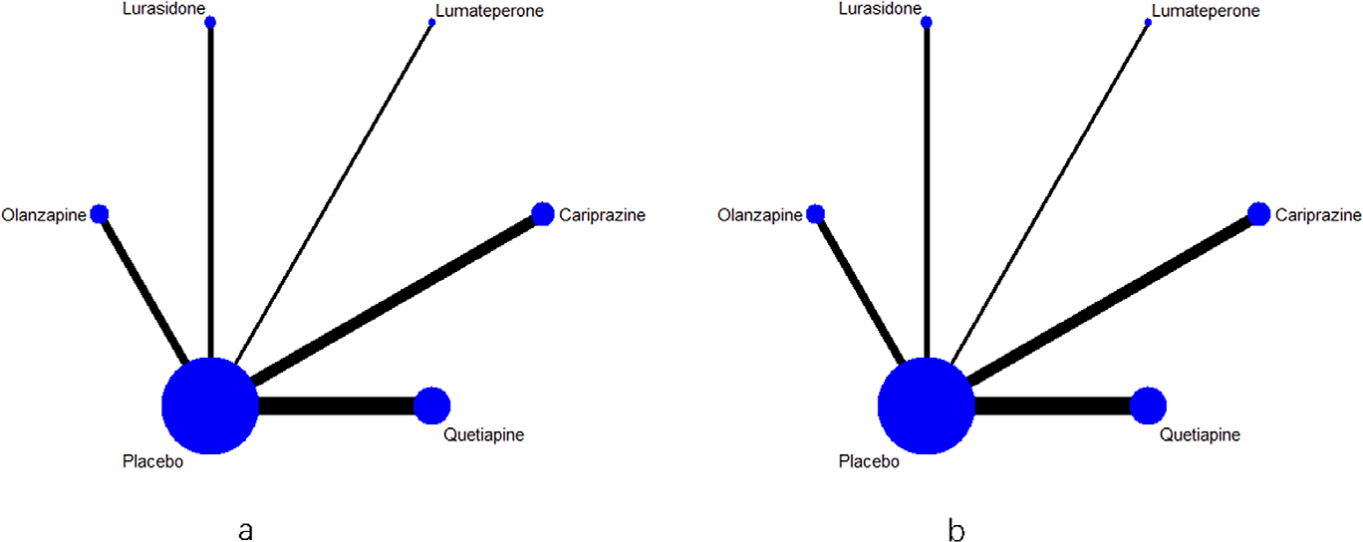
(a) Evidence network for the response rate. (b) Evidence network for the all-cause discontinuation rate.

Figure 2 shows the network plot of six interventions (cariprazine, lumateperone, lurasidone, olanzapine, quetiapine, and placebo) for response rate and all-cause discontinuation. Each connecting line represents treatments that were compared directly in the trial. The size of each node corresponds to the number of studies that relate to a specific treatment, whereas the thickness of each edge corresponds to the number of comparisons contained within the network. As shown in Figure 2, the most common comparison was between placebo and quetiapine.

#### Primary outcomes

For response rate, cariprazine, lumateperone, lurasidone, olanzapine, and quetiapine all showed significantly greater odds of response rate in comparison to the placebo (see Table 3). As shown in the SUCRA rankings (see Table 4 and eFigure 3), quetiapine ranked first followed by lurasidone, lumateperone, olanzapine, and cariprazine when compared to placebo. By pairwise comparison, individuals treated with quetiapine had a more favorable response rate than cariprazine.

**Table 3.** Odds ratios for response (≥50% improvement in MADRS, bottom-left, blue background) and all-cause discontinuation (top-right, yellow background)

Cariprazine	0.94(0.40–1.89)	0.95(0.56–1.50)	0.68(0.43–1.03)	0.96(0.65–1.36)	0.97(0.71–1.30)
0.78(0.47–1.39)	**Lumateperone**	1.15(0.47–2.39)	0.83(0.35–1.67)	1.17(0.52–2.28)	1.18(0.55–2.25)
0.70(0.47–1.09)	0.85(0.50–1.61)	**Lurasidone**	0.75(0.43–1.21)	1.05(0.65–1.61)	1.07(0.71–1.56)
0.91(0.63–1.35)	1.11(0.65–2.00)	1.25(0.83–2.00)	**Olanzapine**	1.45(0.96–2.10)	**1.47(1.04–2.01)**
**0.69(0.51–0.96)**	0.83(0.52–1.47)	0.95(0.66–1.43)	0.74(0.53–1.08)	**Quetiapine**	1.03(0.82–1.27)
**1.45(1.14–1.85)**	**1.75(1.11–2.94)**	**2.00(1.11–2.94)**	**1.54(1.19–2.13)**	**2.08(1.69–2.56)**	**Placebo**

*Note*: Response rate results are on the bottom left, and all-cause discontinuation results are on the top right. Results give the odds ratio [95% credible interval]. The row treatment is the reference treatment.

Bold entries indicate a statistical significance as *P*<0.05.

**Table 4.** Surface under the cumulative ranking curve (SUCRA) for primary and secondary outcomes

Outcome	Carprazine SUCRA (rank)	Lumateperone SUCRA (rank)	Lurasidone SUCRA (rank)	Olanzapine SUCRA (rank)	Quetiapine SUCRA (rank)	Placebo SUCRA (rank)
**Efficacy outcomes**						
Response	0.33(5)	0.63(3)	0.79(2)	0.42(4)	0.84(1)	<0.01(6)
Remission	0.52(3)	0.35(5)	0.74(2)	0.44(4)	0.92(1)	0.03(6)
**Discontinuation outcomes**						
All–cause	0.30(6)	0.53(2)	0.47(3)	0.91(1)	0.42(4)	0.37(5)
Due to adverse events	0.48(4)	0.28(5)	0.63(2)	0.61(3)	0.16(6)	0.84(1)
**Adverse events**						
Somnolence	0.68(3)	0.15(5)	0.74(2)	0.40(4)	0.11(6)	0.91(1)
Headache	0.59(3)	0.08(6)	0.71(2)	0.41(4)	0.85(1)	0.36(5)
Nausea	0.15(6)	0.16(5)	0.36(4)	0.84(2)	0.86(1)	0.63(3)
**Metabolic outcomes**						
≥ 7% weight gain	0.44(3)	1.00(1)	0.35(5)	0.01(6)	0.41(4)	0.79(2)
Change in cholesterol	0.91(1)	0.32(5)	0.56(4)	0.01(6)	0.58(3)	0.62(2)
Change in triglycerides	0.39(4)	0.75(2)	0.77(1)	0.03(6)	0.33(5)	0.74(3)
Change in blood glucose	0.53(3)	0.74(1)	0.42(5)	0.25(6)	0.46(4)	0.61(2)

*Note*: The treatment outcomes of five antipsychotics and placebo were ranked from 1 to 6, (1) indicates the best and (6) indicates the worst performance.

For all-cause discontinuation rates, four AAPs (cariprazine, lumateperone, lurasidone, and quetiapine) were similar to placebo, whereas the rate odds of all-cause discontinuation for olanzapine were lower than placebo (see Table 3). According to the SUCRA rankings (see Table 4 and eFigure 4), olanzapine was the best-tolerated treatment regarding all-cause discontinuation, followed by lumateperone, lurasidone, quetiapine, placebo, and cariprazine.

#### Secondary outcomes

For secondary outcomes, we estimated the remission rate to further evaluate the efficacy. The results showed that cariprazine, lurasidone, olanzapine, and quetiapine (but not lumateperone) had significantly greater odds of remission rate than placebo (see eTable 2). Based on the SUCRA values (see Table 4 and eFigure 5), quetiapine ranked first for remission rate, followed by lurasidone, which was consistent with the results of the response rate.

In terms of discontinuation due to adverse events, quetiapine showed higher odds compared with placebo, whereas others showed similar odds (see eTable 2). According to the SUCRA ranking (see Table 4 and eFigure 6), lurasidone was ranked first as having the highest rate of discontinuation due to adverse events, followed by olanzapine, cariprazine, lumateperone, and quetiapine. As for each adverse event, compared with placebo, cariprazine, and lurasidone demonstrated no significant difference in the rate of somnolence, whereas other AAPs were associated with a greater rate of somnolence than placebo, and lumateperone was associated with higher odds than lurasidone (see eTable 3). According to SUCRA rankings (see Table 4 and eFigure 7), lurasidone was the best-tolerated agent regarding somnolence, with cariprazine, olanzapine, lumateperone, and quetiapine following in the ranking. The incidence of headache for all AAPs was comparable to placebo and quetiapine ranked the best with a lower headache rate, followed by lurasidone (see eTable 3, Table 4, and eFigure 8). Rate of nausea for all AAPs and placebo was comparable, except for cariprazine with a significantly higher odds of nausea than placebo, olanzapine, and quetiapine (see eTable 4). According to SUCRA rankings (see Table 4 and eFigure 9), quetiapine ranked as the best-tolerated treatment with a lower rate of nausea, followed by olanzapine, lurasidone, lumateperone, and cariprazine. For the rate of ≥7% weight gain, lumateperone had significantly lower odds than placebo, whereas cariprazine, olanzapine, and quetiapine were associated with greater odds compared with placebo and no significant difference was observed between lurasidone and placebo (see eTable 4, Table 4, and eFigure 10). No significant difference was observed for change in total cholesterol or triglycerides of all the five AAPs, except for olanzapine showed more increase in total cholesterol and triglycerides than placebo (see eTables 5–6, Table 4 and eFigure 11–12). In addition, all the FDA-approved AAPs showed no difference in change in blood glucose (see eTable 7, Table 4, and eFigure 13).

### Quality evaluation and heterogeneity

**Figure 3.**
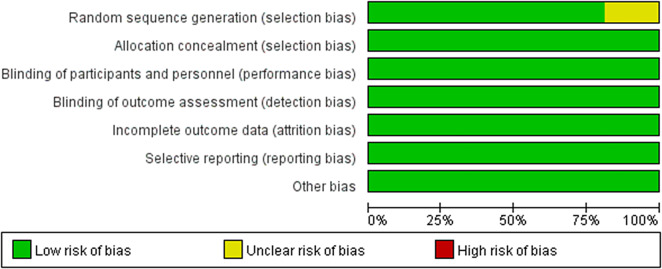
Risk of bias of graph of the included studies.

The quality evaluation showed that the risk of bias was relatively low (see Figure 3), though there were some concerns in the random sequence generation in three studies [11, 20, 25]. The quality of evidence for the primary outcome and remission rate was either highly or moderately reliable for direct comparisons, but less for the NMA evidence. The detailed results for the GRADE assessment are presented in eTable 8a–k. The assessment of transitivity showed that most studies had similar variations in terms of average age, sex, and MARDS score at baseline. Half of these trials enrolled patients exclusively with bipolar I disorder whereas all quetiapine trials included both bipolar I and II patients. Details for results are displayed in Table 2. Heterogeneity assessment showed that the Tau [2] ranged from 0 to 4.59 (see eTable 9), and some studies were considered as moderate heterogeneity.

### Sensitivity analysis and publication bias

Sensitivity analysis did not identify any study that had an excessive influence on the efficacy or safety of AAPs for bipolar depression (see eFigures 14–15). The results of the funnel plot for outcomes are shown in eFigures 16–17. Potential asymmetry could be observed in the funnel plots for the rate of ≥7% weight gain and nausea, suggesting the potential for reporting bias. In addition, Egger’s test showed a significant publication bias for the result of nausea. Detailed results for all the outcomes can be found in eFigures 18–19.

## Discussion

In this study, we conduct an up-to-date NMA of RCTs on the efficacy and tolerability of US FDA-approved five AAPs for acute bipolar depression, including the latest approved agent, lumateperone. In terms of efficacy, all five AAPs had a more favorable treatment response than placebo within a 6- or 8-week monotherapy. Although quetiapine had the highest response and remission rates, it was also the only agent that had a higher likelihood of discontinuation due to adverse events compared to the placebo. Interestingly, olanzapine was the only agent reporting significantly lower odds of all-cause discontinuation compared with the placebo, but was also the only agent that caused a significantly higher increase of total cholesterol and triglycerides than the placebo. There were three AAPs (lumateperone, olanzapine, and quetiapine) that had higher odds of somnolence than the placebo, but only lumateperone had a lower weight gain when compared to the placebo. Other adverse events and metabolic outcomes are variable across different agents. These findings offer an important reference for developing and optimizing individualized pharmacotherapy among adult patients with bipolar depression.

Of the 16 included studies, half of the studies only included individuals with BPAD1, and the remaining half included both BPAD1 and BPAD2. BPAD1 is primarily characterized by overt manic episodes, whereas BPAD2 is characterized by episodes of depression and hypomania, and individuals with BPAD2 experience depressive symptoms more frequently than those with BPAD1. Notably, all six trials of quetiapine included both BPAD1 and BPAD2, and quetiapine was also found to be the most effective drug in the present NMA. Based on these findings, we hypothesize that quetiapine may have a promising effect on patients with BPAD2. Further exploration is warranted to verify this hypothesis.

Compared to the 2020 NMA study [9], the current study enrolled two new RCTs, one for lurasidone and another for lumateperone. In the 2020 NMA study, lurasidone ranked first on change in MADRS score according to the SUCRA rankings [9], whereas the current update study found quetiapine ranked first in terms of the treatment response and remission rates. This gap may be further influenced by high-quality RCTs conducted in the future. Nonetheless, current evidence favors the recommendations in the 2018 CANMAT/ISBD guidelines that quetiapine or lurasidone monotherapy can be the first-line choice for acute bipolar depression [13]. Lumateperone, a recently approved agent, demonstrated a significant improvement in response rate compared to placebo, whereas there was no difference in terms of remission rate. One possible explanation for this discrepancy is that only one study included in the meta-analysis enrolled patients treated with lumateperone. Therefore, the results of the lumateperone subgroup should be interpreted cautiously due to the limited sample size.

Metabolic side effects are a key concern in the clinical use of AAPs. In this study, we found that, following a short-term treatment, olanzapine had much higher odds reaching 33.33 (95% CrI: 12.50–100.00) of >7% weight gain rate compared to placebo, followed by quetiapine (OR: 2.94, 95% CrI: 1.72–5.88), and cariprazine (OR: 2.56, 95% CrI: 1.14–10.00). In addition, olanzapine is the only agent that had a significantly higher change in total cholesterol and triglycerides than the placebo. These findings altogether did not advocate recommending olanzapine as the first-line medication for bipolar patients but still shed light on the potential therapeutic use of olanzapine for individuals with anorexia nervosa [26]. Interestingly, olanzapine was the only agent having significantly lower odds of all-cause discontinuation compared with placebo. This could be an advantage of olanzapine when the patient has a high risk of stopping the medication ahead of schedule.

Sleep disturbance is a prominent symptom in most patients with BD [27]. Refining treatment approaches for co-occurrent sleep disturbance helps to improve mood states and functioning in BD [28]. In the current study, we found that lumateperone, olanzapine, and quetiapine had a more apparent effect of somnolence than placebo, thus showing the potential of being concurrently used as a sleep aid. However, the adverse effects of olanzapine and quetiapine on weight gain limit their clinical application. The newly approved agent, lumateperone, did not deteriorate the metabolic burden in BD patients. Therefore, it could be an advantage of lumateperone for treating bipolar depression with co-occurrent sleep disturbance.

Several major limitations in this NMA study should be mentioned. As an up-to-date NMA, the quality of this study relied on the last comparable systematic review. Transitivity analysis showed that most studies had similar variations in age, sex, and baseline depression severity, but some studies were considered as moderate heterogeneity. Although the demographic profiles between the drug intervention group and the placebo group were largely matched across different RCTs, unidentified confounding factors may still influence the results. In addition, the results from the comparisons between AAPs should be interpreted with caution due to the lack of direct head-to-head comparison studies. Similar to the previous NMA study [9], the current study did not perform meta-regression that can adjust effect modifiers because of a limited number of included trials. The current study included only the MADRS for measuring the severity of depression, whereas other psychometric scales (e.g., the Clinical Global Impression Scale) and other adverse events (e.g., extrapyramidal symptoms and switch to mania) were not analyzed.

## Conclusion

In conclusion, the current NMA provides an up-to-date analysis of the efficacy and tolerability of FDA-approved AAPs for treating adults with acute bipolar depression. All five antipsychotics demonstrated efficacy in treating bipolar depression, with quetiapine and lurasidone showing the most favorable effects. The adverse reactions and metabolic effects of the five agents differ, informing individualized prescriptions of AAPs for treating bipolar depression in clinical practice. More well-designed, high-quality randomized RCTs are needed to consolidate these findings.

## Supporting information

Li et al. supplementary material Li et al. supplementary material

## Supplementary material

The supplementary material for this article can be found at http://doi.org/10.1192/j.eurpsy.2024.25.
