# Supplementary material for: Efficacy and tolerability of FDA-approved atypical antipsychotics for the treatment of bipolar depression: a systematic review and network meta-analysis
Source: Eur Psychiatry. 2024 Mar 15;67(1):e29. doi: 10.1192/j.eurpsy.2024.25 (PMC10988162; doi:10.1192/j.eurpsy.2024.25)
Supplement: Li et al. supplementary material [file S0924933824000257sup001.docx]

**Supplementary materials**

**Search strategy：**

((((((((((((((((((((((((((Bipolar Disorder) OR (Bipolar Disorders)) OR (Disorder, Bipolar)) OR (Affective Psychosis, Bipolar)) OR (Bipolar Affective Psychosis)) OR (Psychoses, Bipolar Affective)) OR (Psychosis, Bipolar Affective)) OR (Manic-Depressive Psychosis)) OR (Manic Depressive Psychosis)) OR (Psychosis, Manic-Depressive)) OR (Psychosis, Manic Depressive)) OR (Bipolar Mood Disorde)) OR (Bipolar Mood Disorders)) OR (Disorder, Bipolar Mood)) OR (Mood Disorder, Bipolar)) OR (Psychoses, Manic-Depressive)) OR (Psychoses, Manic Depressive)) OR (Depression, Bipolar)) OR (Bipolar Depression)) OR (Manic Depression)) OR (Depression, Manic)) OR (Depressions, Manic)) OR (Manic Disorder)) OR (Disorder, Manic)) OR (Manic Disorders) AND (2020/5/1:2022/8/3[pdat])) AND (((((((((((((Cariprazine) ) OR (3-(trans-4-(2-(4-(2,3-dichlorophenyl)piperazin-1-yl)ethyl)cyclohexyl)-1,1-dimethylurea)) OR (Vraylar)) OR (cariprazine hydrochloride)) OR (N'-(trans-4-(2-(4-(2,3-dichlorophenyl)piperazin-1-yl)ethyl)cyclohexyl)-N,N-dimethylurea monohydrochloride)) OR (cariprazine HCl)) OR (trans-4-(2-(4-(2,3-dichlorophenyl)piperazine-1-yl)-ethyl)-N,N-dimethylcarbamoyl-cyclohexyl-amine hydrochloride)) OR (RGH-188) AND (2020/5/1:2022/8/3[pdat])) OR ((((((((((((((Lurasidone Hydrochloride) OR (Hydrochloride, Lurasidone)) OR (Lurasidone HCl)) OR (HCl, Lurasidone)) OR (SM 13496)) OR (13496, SM)) OR (SM13496)) OR (SM-13,496)) OR (SM 13,496)) OR (SM13,496)) OR (SM-13496)) OR (Lurasidone)) OR (N-(2-(4-(1,2-benzisothiazol-3-yl)-1-piperazinylmethyl)-1-cyclohexylmethyl)-2,3 bicyclo(2.2.1)heptanedicarboximide)) OR (Latuda) AND (2020/5/1:2022/8/3[pdat]))) OR (((((((((Olanzapine) OR (Olanzapine)) OR (LY-170052)) OR (LY 170052)) OR (LY170052)) OR (Zyprexa)) OR (Zolafren)) OR (LY 170053)) OR (Olanzapine Pamoate) AND (2020/5/1:2022/8/3[pdat]))) OR (((((((((Quetiapine Fumarate) OR (Ethanol, 2-(2-(4-dibenzo(b,f)(1,4)thiazepin-11-yl-1-piperazinyl)ethoxy)-, (E)-2-butenedioate (2:1) (salt))) OR (Seroquel)) OR (ICI 204,636)) OR (ICI-204636)) OR (ICI204636)) OR (ICI 204636)) OR (Quetiapine)) OR (2-(2-(4-dibenzo(b,f)(1,4)thiazepine-11-yl-1-piperazinyl)ethoxy)ethanol) AND (2020/5/1:2022/8/3[pdat]))) OR ((((lumateperone) OR (ITI-007)) OR (ITI-722)) OR (lumateperone tosylate) AND (2020/5/1:2022/8/3[pdat])) AND (2020/5/1:2022/8/3[pdat]))) AND ("randomized controlled trial"[pt] OR "controlled clinical trial"[pt] OR randomized[tiab] OR placebo[tiab] OR "drug therapy"[sh] OR randomly[tiab] OR trial[tiab] OR groups[tiab] AND (2020/5/1:2022/8/3[pdat]))

| FDA-approved antipsychotics | Depressive episode | Manic episode or mixed features | Acute treatment | Maintenance treatment | Adult | Child |
| --- | --- | --- | --- | --- | --- | --- |
| quetiapine | √ | √ | √ | Immediate release: √  Extended release: × | √ | √ |
| olanzapine-fluoxetine | √ | × | √ | × | √ | √ |
| lurasidone | √ | × | √ | × | √ | √ |
| cariprazine | √ | √ | √ | × | √ | × |
| lumateperone | √ | × | √ | × | √ | × |

**eTable 1.** FDA-approved antipsychotics for the management of bipolar disorder.

**eTable 2.** Odds ratios for remission (MADRS≤12 or 10) and discontinuation due to adverse events

Note: Remission rate results are on the bottom left, and discontinuation due to the adverse events results are on the top-right. Results give the odds ratio [95% credible interval]. The row treatment is the reference treatment.

**eTable 3.** Odds ratios for somnolence and headache

Note: Somnolence rate results are on the bottom left, and headache rate results are on the top-right. Results give the odds ratio [95% credible interval]. The row treatment is the reference treatment.

**eTable 4.** Odds ratios for ≥7% weight gain and nausea

Note: ≥7% weight gain rate results are on the bottom left, and nausea rate results are on the top-right. Results give the odds ratio [95% credible interval]. The row treatment is the reference treatment.

**eTable 5.** Change from baseline in total cholesterol

Note: Results give the odds ratio [95% credible interval]. The row treatment is the reference treatment.

**eTable 6.** Change from baseline in triglycerides

Note: Results give the odds ratio [95% credible interval]. The row treatment is the reference treatment.

**eTable 7.** Change from baseline in blood glucose

Note: Results give the odds ratio [95% credible interval]. The row treatment is the reference treatment.

**eTable 8a.** GRADE assessment of the response rate

| Comparison | Direct evidence | | | Network meta-analysis | |
| --- | --- | --- | --- | --- | --- |
|  | Number of studies | Odds ratio (95% confidence interval) | Quality of evidence | Odds ratio (95% credible interval) | Quality of evidence |
| Cariprazine vs placebo | 4 | 1.46 [1.18, 1.80] | Moderate^%^ | 1.45 [1.14, 1.85] | Moderate^%^ |
| Cariprazine vs  Lumateperone | 0 |  |  | 0.78 [0.47, 1.39] | Low^#%^ |
| Cariprazine vs  Lurasidone | 0 |  |  | 0.70 [0.47, 1.09] | Low^#%^ |
| Cariprazine vs  Olanzapine | 0 |  |  | 0.91 [0.63, 1.35] | Low^#%^ |
| Cariprazine vs  Quetiapine | 0 |  |  | 0.69 [0.51, 0.96] | Moderate^#^ |
| Lumateperone vs placebo | 1 | 1.80 [1.19, 2.72] | Moderate^%^ | 1.75 [1.11, 2.94] | Moderate^%^ |
| Lumateperone vs  Lurasidone | 0 |  |  | 0.85 [0.50, 1.61] | Very low^#%^^ |
| Lumateperone vs  Olanzapine | 0 |  |  | 1.11 [0.65, 2.00] | Low^#%^ |
| Lumateperone vs  Quetiapine | 0 |  |  | 0.83 [0.52, 1.47] | Low^#%^ |
| Lurasidone vs placebo | 2 | 2.01 [1.53, 2.66] | Moderate^%^ | 2.00 [1.11, 2.94] | Moderate^%^ |
| Lurasidone vs Olanzapine | 0 |  |  | 1.25 [0.83, 2.00] | Low^#%^ |
| Lurasidone vs Quetiapine | 0 |  |  | 0.95 [0.66, 1.43] | Low^#%^ |
| Olanzapine vs placebo | 3 | 1.53 [1.21, 1.93] | Moderate^%^ | 1.54 [1.19, 2.13] | Moderate^%^ |
| Olanzapine vs Quetiapine | 0 |  |  | 0.74 [0.53, 1.08] | Low^#%^ |
| Quetiapine vs placebo | 6 | 2.07 [1.75, 2.46] | Moderate^%^ | 2.08 [1.69, 2.56] | Moderate^%^ |

Factors downgrading any specific evidence: * Limitations (risk of bias), $ Inconsistency of results, # indirectness of results, % Imprecision, ^ Publications bias

**eTable 8b.** GRADE assessment of all-cause discontinuation

| Comparison | Direct evidence | | | Network meta-analysis | |
| --- | --- | --- | --- | --- | --- |
|  | Number of studies | Odds ratio (95% confidence interval) | Quality of evidence | Odds ratio (95% credible interval) | Quality of evidence |
| Cariprazine vs placebo | 4 | 1.03 [0.81, 1.32] | Moderate^%^ | 1.03 [0.78, 1.41] | Moderate^%^ |
| Cariprazine vs  Lumateperone | 0 |  |  | 1.06 [0.53,2.50] | Low^#%^ |
| Cariprazine vs  Lurasidone | 0 |  |  | 1.05 [0.67, 1.79] | Low^#%^ |
| Cariprazine vs  Olanzapine | 0 |  |  | 1.47 [0.97, 2.33] | Low^#%^ |
| Cariprazine vs  Quetiapine | 0 |  |  | 1.04 [0.74, 1.54] | Moderate^#^ |
| Lumateperone vs placebo | 1 | 0.91 [0.48,1.70] | Moderate^%^ | 0.85 [0.44, 1.82] | Moderate^%^ |
| Lumateperone vs  Lurasidone | 0 |  |  | 0.87 [0.42, 2.13] | Very low^#%^^ |
| Lumateperone vs  Olanzapine | 0 |  |  | 1.20 [0.60, 2.86] | Low^#%^ |
| Lumateperone vs  Quetiapine | 0 |  |  | 0.85 [0.44, 1.92] | Low^#%^ |
| Lurasidone vs placebo | 2 | 0.96 [0.70, 1.31] | Moderate^%^ | 0.93 [0.65, 1.43] | Moderate^%^ |
| Lurasidone vs Olanzapine | 0 |  |  | 1.33 [0.83, 2.33] | Low^#%^ |
| Lurasidone vs Quetiapine | 0 |  |  | 0.95 [0.62, 1.54] | Low^#%^ |
| Olanzapine vs placebo | 3 | 0.68 [0.54, 0.86] | High | 0.68 [0.50, 0.96] | Moderate^#^ |
| Olanzapine vs Quetiapine | 0 |  |  | 0.69 [0.48, 1.04] | Low^#%^ |
| Quetiapine vs placebo | 6 | 0.99 [0.83, 1.17] | Moderate^%^ | 0.97 [0.79, 1.22] | Moderate^%^ |

Factors downgrading any specific evidence: * Limitations (risk of bias), $ Inconsistency of results, # indirectness of results, % Imprecision, ^ Publications bias

**eTable 8c.** GRADE assessment of the remission rate

| Comparison | Direct evidence | | | Network meta-analysis | |
| --- | --- | --- | --- | --- | --- |
|  | Number of studies | Odds ratio (95% confidence interval) | Quality of evidence | Odds ratio (95% credible interval) | Quality of evidence |
| Cariprazine vs placebo | 4 | 1.53 [1.21, 1.94] | Moderate^%^ | 1.52 [1.16, 2.04] | Moderate^%^ |
| Cariprazine vs  Lumateperone | 0 |  |  | 1.11 [0.65,2.08] | Low^#%^ |
| Cariprazine vs  Lurasidone | 0 |  |  | 0.83 [0.54, 1.35] | Low^#%^ |
| Cariprazine vs  Olanzapine | 0 |  |  | 1.04 [0.69, 1.59] | Low^#%^ |
| Cariprazine vs  Quetiapine | 0 |  |  | 0.75 [0.54, 1.06] | Moderate^#^ |
| Lumateperone vs placebo | 1 | 1.32 [0.86,2.01] | Moderate^%^ | 1.28 [0.79, 2.17] | Moderate^%^ |
| Lumateperone vs  Lurasidone | 0 |  |  | 0.70 [0.39, 1.37] | Very low^#%^^ |
| Lumateperone vs  Olanzapine | 0 |  |  | 0.87 [0.50, 1.64] | Low^#%^ |
| Lumateperone vs  Quetiapine | 0 |  |  | 0.63 [0.38, 1.12] | Low^#%^ |
| Lurasidone vs placebo | 2 | 1.79 [1.33, 2.40] | Moderate^%^ | 1.75 [1.25, 2.56] | Moderate^%^ |
| Lurasidone vs Olanzapine | 0 |  |  | 1.20 [0.77, 2.00] | Low^#%^ |
| Lurasidone vs Quetiapine | 0 |  |  | 0.87 [0.50, 1.04] | Low^#%^ |
| Olanzapine vs placebo | 3 | 1.41 [1.11, 1.80] | Moderate^%^ | 1.43 [1.08, 2.00] | Moderate^%^ |
| Olanzapine vs Quetiapine | 0 |  |  | 0.70 [0.50, 1.04] | Low^#%^ |
| Quetiapine vs placebo | 6 | 2.03 [1.71, 2.40] | Moderate^%^ | 2.00 [1.64, 2.50] | Moderate^%^ |

Factors downgrading any specific evidence: * Limitations (risk of bias), $ Inconsistency of results, # indirectness of results, % Imprecision, ^ Publications bias

**eTable 8d.** GRADE assessment of discontinuation due to adverse events

| Comparison | Direct evidence | | | Network meta-analysis | |
| --- | --- | --- | --- | --- | --- |
|  | Number of studies | Odds ratio (95% confidence interval) | Quality of evidence | Odds ratio (95% credible interval) | Quality of evidence |
| Cariprazine vs placebo | 4 | 1.36 [0.86, 2.13] | Moderate^%^ | 1.45 [0.74, 3.70] | Low^#%^ |
| Cariprazine vs  Lumateperone | 0 |  |  | 0.41 [0.10,4.17] | Low^#%^ |
| Cariprazine vs  Lurasidone | 0 |  |  | 1.01 [0.35, 5.00] | Low^#%^ |
| Cariprazine vs  Olanzapine | 0 |  |  | 1.03 [0.41, 4.76] | Low^#%^ |
| Cariprazine vs  Quetiapine | 0 |  |  | 0.55 [0.23, 1.75] | Low^#%^ |
| Lumateperone vs placebo | 1 | 2.29 [0.78,6.71] | Moderate^%^ | 1.67 [0.48,12.50] | Low^#%^ |
| Lumateperone vs  Lurasidone | 0 |  |  | 0.70 [0.39, 1.37] | Very low^#%^^ |
| Lumateperone vs  Olanzapine | 0 |  |  | 0.87 [0.50, 1.64] | Low^#%^ |
| Lumateperone vs  Quetiapine | 0 |  |  | 0.63 [0.38, 1.12] | Low^#%^ |
| Lurasidone vs placebo | 2 | 1.20 [0.68, 2.12] | Moderate^%^ | 1.09 [0.44, 3.57] | Moderate^%^ |
| Lurasidone vs Olanzapine | 0 |  |  | 0.78 [0.27, 4.17] | Low^#%^ |
| Lurasidone vs Quetiapine | 0 |  |  | 0.40 [0.14, 1.62] | Low^#%^ |
| Olanzapine vs placebo | 3 | 1.45 [0.94, 2.23] | Moderate^%^ | 1.14 [0.47, 2.94] | Moderate^%^ |
| Olanzapine vs Quetiapine | 0 |  |  | 0.42 [0.15, 1.37] | Low^#%^ |
| Quetiapine vs placebo | 6 | 2.30 [1.67, 3.18] | High | 2.38 [1.41, 5.00] | Moderate^%^ |

Factors downgrading any specific evidence: * Limitations (risk of bias), $ Inconsistency of results, # indirectness of results, % Imprecision, ^ Publications bias

**eTable 8e.** GRADE assessment of the rate of somnolence

| Comparison | Direct evidence | | | Network meta-analysis | |
| --- | --- | --- | --- | --- | --- |
|  | Number of studies | Odds ratio (95% confidence interval) | Quality of evidence | Odds ratio (95% credible interval) | Quality of evidence |
| Cariprazine vs placebo | 1 | 1.42 [0.57, 3.53] | Moderate^%^ | 1.28 [0.51, 5.00] | Moderate^%^ |
| Cariprazine vs  Lumateperone | 0 |  |  | 0.24 [0.08,1.43] | Low^#%^ |
| Cariprazine vs  Lurasidone | 0 |  |  | 0.88 [0.29, 4.55] | Low^#%^ |
| Cariprazine vs  Olanzapine | 0 |  |  | 0.42 [0.15, 2.00] | Low^#%^ |
| Cariprazine vs  Quetiapine | 0 |  |  | 0.26 [0.10, 1.09] | Moderate^#^ |
| Lumateperone vs placebo | 1 | 4.58 [2.39,8.76] | Moderate^%^ | 4.17 [1.92,12.50] | Low^#%^ |
| Lumateperone vs  Lurasidone | 0 |  |  | 2.86 [1.06, 12.50] | Very low^#%^^ |
| Lumateperone vs  Olanzapine | 0 |  |  | 1.37 [0.56, 5.00] | Low^#%^ |
| Lumateperone vs  Quetiapine | 0 |  |  | 0.85 [0.36, 2.70] | Low^#%^ |
| Lurasidone vs placebo | 2 | 1.29 [0.69, 2.43] | Moderate^%^ | 1.23 [0.62, 2.94] | Moderate^%^ |
| Lurasidone vs Olanzapine | 0 |  |  | 0.41 [0.18, 1.23] | Low^#%^ |
| Lurasidone vs Quetiapine | 0 |  |  | 0.25 [0.11, 0.67] | Low^#%^ |
| Olanzapine vs placebo | 2 | 2.81 [2.02, 3.91] | High | 2.78[1.61, 5.26] | Moderate^ |
| Olanzapine vs Quetiapine | 0 |  |  | 0.55 [0.29, 1.20] | Low^#%^ |
| Quetiapine vs placebo | 6 | 4.61 [3.25, 6.54] | High | 4.76 [3.33, 7.14] | Moderate^%^ |

Factors downgrading any specific evidence: * Limitations (risk of bias), $ Inconsistency of results, # indirectness of results, % Imprecision, ^ Publications bias

**eTable 8f.** GRADE assessment of the rate of headache

| Comparison | Direct evidence | | | Network meta-analysis | |
| --- | --- | --- | --- | --- | --- |
|  | Number of studies | Odds ratio (95% confidence interval) | Quality of evidence | Odds ratio (95% credible interval) | Quality of evidence |
| Cariprazine vs placebo | 3 | 0.77 [0.51, 1.16] | Moderate^%^ | 0.75 [0.40, 1.67] | Moderate^%^ |
| Cariprazine vs  Lumateperone | 0 |  |  | 0.32 [0.11, 1.54] | Low^#%^ |
| Cariprazine vs  Lurasidone | 0 |  |  | 1.00 [0.40, 3.70] | Low^#%^ |
| Cariprazine vs  Olanzapine | 0 |  |  | 0.62 [0.20, 2.08] | Low^#%^ |
| Cariprazine vs  Quetiapine | 0 |  |  | 1.20 [0.55, 3.23] | Moderate^#^ |
| Lumateperone vs placebo | 1 | 1.98 [1.23,3.19] | Moderate^%^ | 1.69 [0.65,6.25] | Very low^#%^^ |
| Lumateperone vs  Lurasidone | 0 |  |  | 2.27 [0.74, 12.50] | Low^#%^ |
| Lumateperone vs  Olanzapine | 0 |  |  | 1.35 [0.37, 6.67] | Low^#%^ |
| Lumateperone vs  Quetiapine | 0 |  |  | 2.70 [0.94, 11.11] | Low^#%^ |
| Lurasidone vs placebo | 2 | 0.67 [0.31, 1.46] | Moderate^%^ | 0.62 [0.28, 1.56] | Moderate^%^ |
| Lurasidone vs Olanzapine | 0 |  |  | 0.50 [0.15, 1.92] | Low^#%^ |
| Lurasidone vs Quetiapine | 0 |  |  | 1.00 [0.40, 3.03] | Low^#%^ |
| Olanzapine vs placebo | 2 | 1.57 [0.21, 11.82] | Moderate^%^ | 0.95[0.49, 3.13] | Low^#%^ |
| Olanzapine vs Quetiapine | 0 |  |  | 1.53 [0.70, 7.14] | Low^#%^ |
| Quetiapine vs placebo | 5 | 0.58 [0.44, 0.75] | Moderate^%^ | 0.58 [0.36, 1.02] | Moderate^%^ |

Factors downgrading any specific evidence: * Limitations (risk of bias), $ Inconsistency of results, # indirectness of results, % Imprecision, ^ Publications bias

**eTable 8g.** GRADE assessment of the rate of nausea

| Comparison | Direct evidence | | | Network meta-analysis | |
| --- | --- | --- | --- | --- | --- |
|  | Number of studies | Odds ratio (95% confidence interval) | Quality of evidence | Odds ratio (95% credible interval) | Quality of evidence |
| Cariprazine vs placebo | 4 | 2.46 [1.42, 4.25] | Moderate^%^ | 2.86 [1.52, 6,67] | Moderate^%^ |
| Cariprazine vs  Lumateperone | 0 |  |  | 0.58 [0.16, 4.76] | Low^#%^ |
| Cariprazine vs  Lurasidone | 0 |  |  | 1.43 [0.56, 5.26] | Low^#%^ |
| Cariprazine vs  Olanzapine | 0 |  |  | 3.45 [1.20, 14.29] | Low^#%^ |
| Cariprazine vs  Quetiapine | 0 |  |  | 1.20 [0.55, 3.23] | Moderate^#^ |
| Lumateperone vs placebo | 1 | 3.15 [1.00, 9.96] | Moderate^%^ | 2.50 [0.77,16.67] | Low^#%^ |
| Lumateperone vs  Lurasidone | 0 |  |  | 1.25 [0.34, 11.11] | low^#%^ |
| Lumateperone vs  Olanzapine | 0 |  |  | 1.35 [0.37, 6.67] | Low^#%^ |
| Lumateperone vs  Quetiapine | 0 |  |  | 2.94 [0.80, 33.33] | Low^#%^ |
| Lurasidone vs placebo | 2 | 1.78 [1.10, 2.90] | Moderate^%^ | 1.67 [0.78, 4.17] | Moderate^%^ |
| Lurasidone vs Olanzapine | 0 |  |  | 2.00 [0.61, 8.33] | Low^#%^ |
| Lurasidone vs Quetiapine | 0 |  |  | 2.22 [0.92, 6.67] | Low^#%^ |
| Olanzapine vs placebo | 2 | 0.94 [0.17, 5.13] | Moderate^%^ | 0.62 [0.29, 2.13] | Moderate^%^ |
| Olanzapine vs Quetiapine | 0 |  |  | 0.85 [0.35, 3.45] | Low^#%^ |
| Quetiapine vs placebo | 5 | 0.69 [0.46, 1.03] | Moderate^%^ | 0.68 [0.42, 1.25] | Moderate^%^ |

Factors downgrading any specific evidence: * Limitations (risk of bias), $ Inconsistency of results, # indirectness of results, % Imprecision, ^ Publications bias

**eTable 8h.** GRADE assessment of the rate of ≥ 7% weight gain

| Comparison | Direct evidence | | | Network meta-analysis | |
| --- | --- | --- | --- | --- | --- |
|  | Number of studies | Odds ratio (95% confidence interval) | Quality of evidence | Odds ratio (95% credible interval) | Quality of evidence |
| Cariprazine vs placebo | 4 | 1.99 [0.84, 4.70] | Moderate^%^ | 2.56 [1.14, 10.00] | Low^#%^ |
| Cariprazine vs  Lumateperone | 0 |  |  | 20.00 [3.39, 3.79E+4] | Low^#%^ |
| Cariprazine vs  Lurasidone | 0 |  |  | 0.35 [0.07, 5.26] | Low^#%^ |
| Cariprazine vs  Olanzapine | 0 |  |  | 0.05 [0.01, 0.41] | Low^#%^ |
| Cariprazine vs  Quetiapine | 0 |  |  | 0.79 [0.31, 3.57] | Low^#%^ |
| Lumateperone vs placebo | 1 | 0.09 [0.00, 1.64] | Moderate^%^ | 0.00 [0.00, 0.74] | Low^#%^ |
| Lumateperone vs  Lurasidone | 0 |  |  | 0.00 [0.00, 0.30] | Very low^#%^^ |
| Lumateperone vs  Olanzapine | 0 |  |  | 0.00 [0.00, 0.03] | Low^#%^ |
| Lumateperone vs  Quetiapine | 0 |  |  | 0.00 [0.00, 0.25] | Low^#%^ |
| Lurasidone vs placebo | 2 | 4.08 [0.74, 22.30] | Moderate^%^ | 2.94 [0.85, 33.33] | Low^#^^ |
| Lurasidone vs Olanzapine | 0 |  |  | 0.06 [0.01, 1.22] | Low^#%^ |
| Lurasidone vs Quetiapine | 0 |  |  | 0.88 [0.24, 11.11] | Low^#%^ |
| Olanzapine vs placebo | 2 | 36.27 [11.46, 114.86] | Moderate^%^ | 33.33 [12.50, 100.00] | Moderate^%^ |
| Olanzapine vs Quetiapine | 0 |  |  | 9.09 [3.13, 50.00] | Low^#%^ |
| Quetiapine vs placebo | 6 | 3.11 [1.92, 5.04] | Moderate^%^ | 2.94 [1.72, 5.88] | Moderate^%^ |

Factors downgrading any specific evidence: * Limitations (risk of bias), $ Inconsistency of results, # indirectness of results, % Imprecision, ^ Publications bias

**eTable 8i.** GRADE assessment of change from baseline in total cholesterol

| Comparison | Direct evidence | | | Network meta-analysis | |
| --- | --- | --- | --- | --- | --- |
|  | Number of studies | Odds ratio (95% confidence interval) | Quality of evidence | Odds ratio (95% credible interval) | Quality of evidence |
| Cariprazine vs placebo | 4 | -2.72 [-5.76, 0.31] | Moderate^%^ | -2.66 [-6.75, 1.53] | Moderate^%^ |
| Cariprazine vs  Lumateperone | 0 |  |  | -7.36 [-17.99, 3.38] | Low^#%^ |
| Cariprazine vs  Lurasidone | 0 |  |  | -3.32 [-10.09, 3.60] | Low^#%^ |
| Cariprazine vs  Olanzapine | 0 |  |  | -15.54 [-22.60, 9.20] | Low^#%^ |
| Cariprazine vs  Quetiapine | 0 |  |  | -3.15 [-9.66, 3.39] | Moderate^#^ |
| Lumateperone vs placebo | 1 | 4.70 [-3.49, 12.89] | Moderate^%^ | 4.70 [-5.18, 14.57] | Moderate^%^ |
| Lumateperone vs  Lurasidone | 0 |  |  | 4.03 [-7.25, 15.32] | Very low^#%^^ |
| Lumateperone vs  Olanzapine | 0 |  |  | -8.18 [-19.63, 2.74] | Low^#%^ |
| Lumateperone vs  Quetiapine | 0 |  |  | 4.21 [-6.92, 15.26] | Low^#%^ |
| Lurasidone vs placebo | 2 | 0.69 [-3.09, 4.47] | Moderate^%^ | 0.67 [-4.81, 6.10] | Moderate^%^ |
| Lurasidone vs Olanzapine | 0 |  |  | -12.22 [-20.28, -5.02] | Low^#%^ |
| Lurasidone vs Quetiapine | 0 |  |  | 0.17 [-7.27, 7.54] | Low^#%^ |
| Olanzapine vs placebo | 3 | 13.04 [7.64, 18.45] | Moderate^%^ | 12.88 [8.03, 18.61] | Moderate^%^ |
| Olanzapine vs Quetiapine | 0 |  |  | 12.39 [5.46, 20.02] | Low^#%^ |
| Quetiapine vs placebo | 3 | 0.55 [-5.10, 6.19] | Moderate^%^ | 0.50 [-4.52, 5.56] | Moderate^%^ |

Factors downgrading any specific evidence: * Limitations (risk of bias), $ Inconsistency of results, # indirectness of results, % Imprecision, ^ Publications bias

**eTable 8j.** GRADE assessment of change from baseline in triglycerides

| Comparison | Direct evidence | | | Network meta-analysis | |
| --- | --- | --- | --- | --- | --- |
|  | Number of studies | Odds ratio (95% confidence interval) | Quality of evidence | Odds ratio (95% credible interval) | Quality of evidence |
| Cariprazine vs placebo | 4 | 8.17 [1.53, 14.82] | Moderate^%^ | 8.28 [-1.99, 18.88] | Low^#%^ |
| Cariprazine vs  Lumateperone | 0 |  |  | 10.37 [-13.69, 34.71] | Low^#%^ |
| Cariprazine vs  Lurasidone | 0 |  |  | 9.73 [-8.04, 27.45] | Low^#%^ |
| Cariprazine vs  Olanzapine | 0 |  |  | -21.34 [-46.05, 0.61] | Low^#%^ |
| Cariprazine vs  Quetiapine | 0 |  |  | -2.93 [-23.04, 16.81] | Moderate^#^ |
| Lumateperone vs placebo | 1 | -2.00 [-17.55, 13.55] | Moderate^%^ | -2.09 [-23.92, 19.88] | Moderate^%^ |
| Lumateperone vs  Lurasidone | 0 |  |  | -0.64 [-27.08, 25.43] | Very low^#%^^ |
| Lumateperone vs  Olanzapine | 0 |  |  | -31.71 [-63.68, -2.87] | Low^#%^ |
| Lumateperone vs  Quetiapine | 0 |  |  | -13.29 [-41.46, 14.12] | Low^#%^ |
| Lurasidone vs placebo | 2 | -1.72 [-10.72, 7.28] | Moderate^%^ | -1.45 [-15.76, 13.1] | Moderate^%^ |
| Lurasidone vs Olanzapine | 0 |  |  | -31.07 [-57.98, -7.00] | Low^#%^ |
| Lurasidone vs Quetiapine | 0 |  |  | -12.66 [-35.09, 9.43] | Low^#%^ |
| Olanzapine vs placebo | 2 | 27.71 [11.61, 43.81] | Moderate^%^ | 29.62 [9.97, 51.99] | Moderate^%^ |
| Olanzapine vs Quetiapine | 0 |  |  | 18.42 [-7.29, 46.49] | Low^#%^ |
| Quetiapine vs placebo | 3 | 10.84 [-3.27, 24.95] | Moderate^%^ | 11.20 [-5.69, 28.48] | Moderate^%^ |

Factors downgrading any specific evidence: * Limitations (risk of bias), $ Inconsistency of results, # indirectness of results, % Imprecision, ^ Publications bias

**eTable 8k.** GRADE assessment of change from baseline in blood glucose

| Comparison | Direct evidence | | | Network meta-analysis | |
| --- | --- | --- | --- | --- | --- |
|  | Number of studies | Odds ratio (95% confidence interval) | Quality of evidence | Odds ratio (95% credible interval) | Quality of evidence |
| Cariprazine vs placebo | 4 | 0.32 [-1.29, 1.94] | Moderate^%^ | 0.23 [-2.89, 3.28] | Moderate^%^ |
| Cariprazine vs  Lumateperone | 0 |  |  | 1.71 [-5.31, 8.65] | Low^#%^ |
| Cariprazine vs  Lurasidone | 0 |  |  | -0.55 [-5.73, 4.73] | Low^#%^ |
| Cariprazine vs  Olanzapine | 0 |  |  | -1.46 [-6.29, 3.16] | Low^#%^ |
| Cariprazine vs  Quetiapine | 0 |  |  | -0.33 [-4.45, 3.66] | Moderate^#^ |
| Lumateperone vs placebo | 1 | -1.50 [-4.97, 1.97] | Moderate^%^ | -1.48 [-7.70, 4.81] | Moderate^%^ |
| Lumateperone vs Lurasidone | 0 |  |  | -2.26 [-9.81, 5.36] | Very low^#%^^ |
| Lumateperone vs  Olanzapine | 0 |  |  | -3.17 [-10.42, 4.02] | Low^#%^ |
| Lumateperone vs  Quetiapine | 0 |  |  | -2.04 [-8.83, 4.75] | Low^#%^ |
| Lurasidone vs placebo | 2 | 1.10 [-0.90, 3.10] | Moderate^%^ | 0.78 [-3.50, 4.96] | Moderate^%^ |
| Lurasidone vs Olanzapine | 0 |  |  | -0.91 [-6.58, 4.53] | Low^#%^ |
| Lurasidone vs Quetiapine | 0 |  |  | 0.22 [-4.87, 5.13] | Low^#%^ |
| Olanzapine vs placebo | 3 | 1.47 [-0.27, 3.21] | Moderate^%^ | 1.69 [-1.86, 5.35] | Moderate^%^ |
| Olanzapine vs Quetiapine | 0 |  |  | 1.14 [-3.28, 5.62] | Low^#%^ |
| Quetiapine vs placebo | 6 | 0.39 [-1.13, 1.90] | Moderate^%^ | 0.55[-2.05, 3.23] | Moderate^%^ |

Factors downgrading any specific evidence: * Limitations (risk of bias), $ Inconsistency of results, # indirectness of results, % Imprecision, ^ Publications bias

**eTable 9.** Heterogeneity Assessment through Tau^2^ of the networks

| **Outcomes** | **Between study variance Tau^2^** |
| --- | --- |
| Odds Ratio for response | 0 |
| Odds Ratios for all-cause discontinuation | 0.12 |
| Odds Ratios for remission (MADRS≤ 12 or≤ 10) | 0.10 |
| Odds Ratios for discontinuation due to adverse events | 0.51 |
| Odds Ratios for somnolence | 0.21 |
| Odds Ratios for headache | 0.39 |
| Odds Ratio for nausea | 0.35 |
| Odds Ratios of ≥7% weight gain | 0.33 |
| Change from Baseline in total cholesterol | 1.74 |
| Change from Baseline in triglycerides | 4.59 |
| Change from Baseline in blood glucose | 2.29 |

| **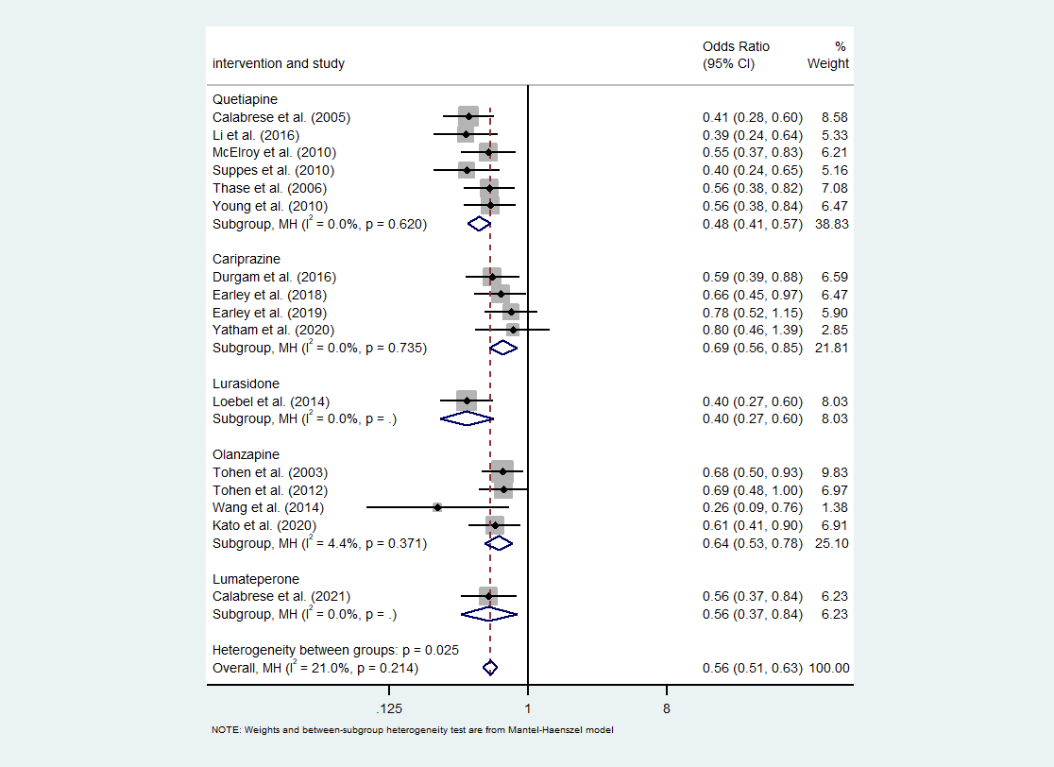 Fig. 1a.** Forest plot for the response rate using a fixed effects model. The Q test and *I^2^* statistic were used to test the heterogeneity. CI, confidence interval. | 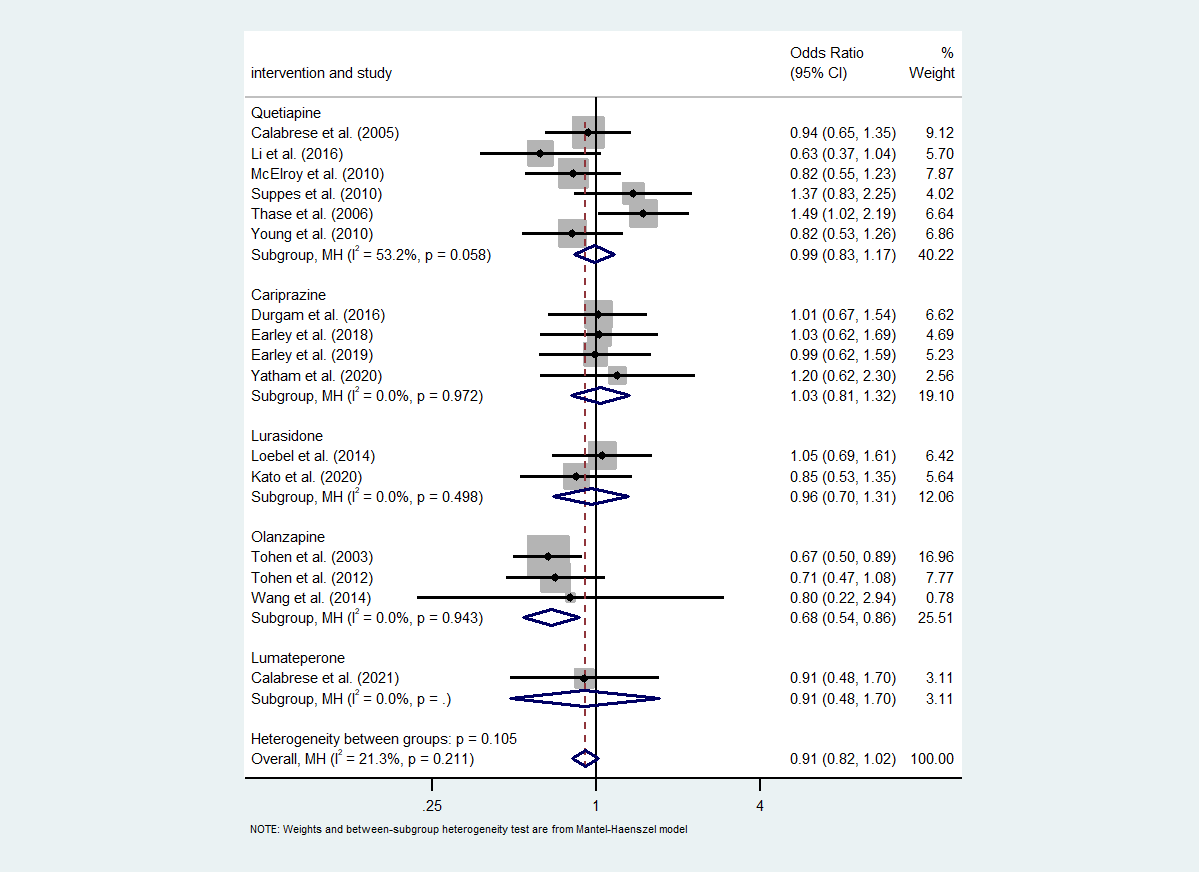**eFig. 1b.** Forest plot for the rate of all-cause discontinuation using a fixed effects model. The Q test and *I^2^* statistic were used to test the heterogeneity. CI, confidence interval. |
| --- | --- |

| 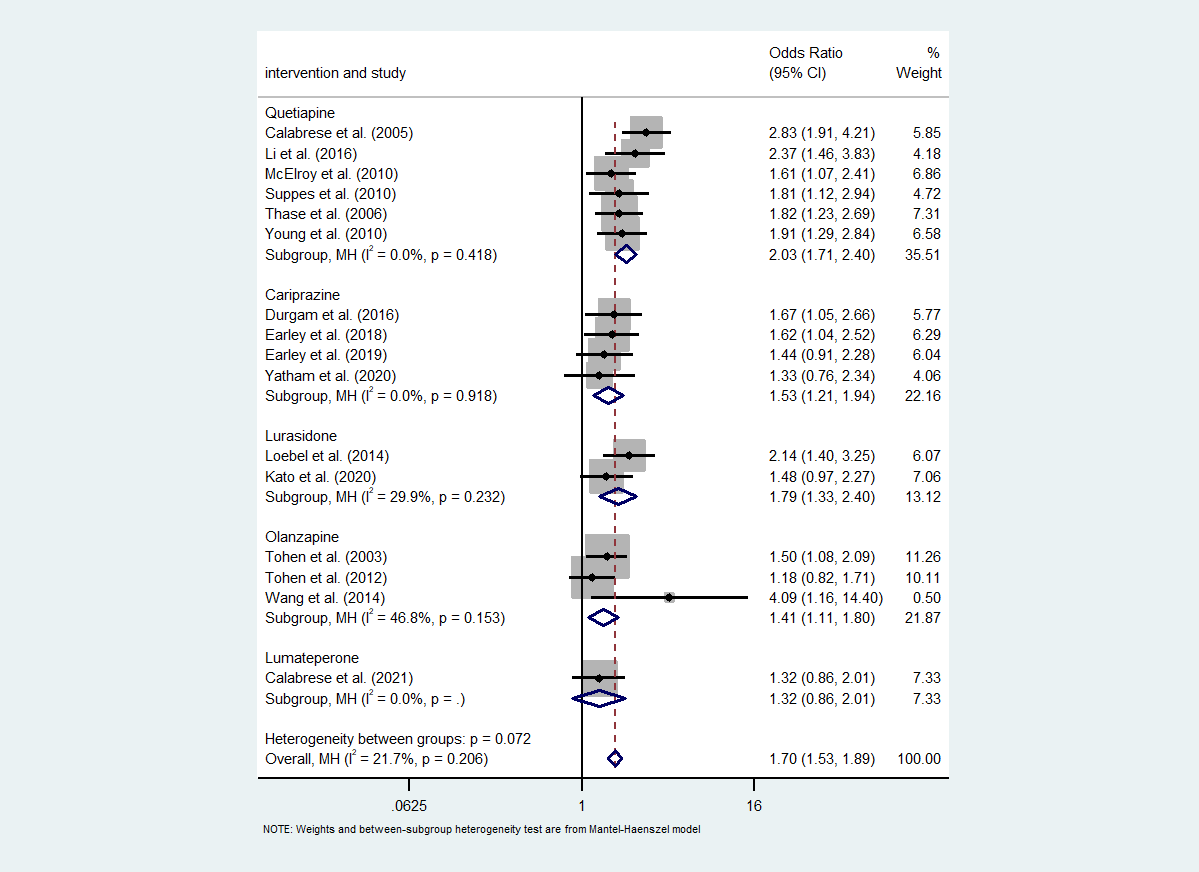**eFig. 1c.** Forest plot for the remission rate using a fixed effects model. The Q test and *I^2^* statistic were used to test the heterogeneity. CI, confidence interval. | 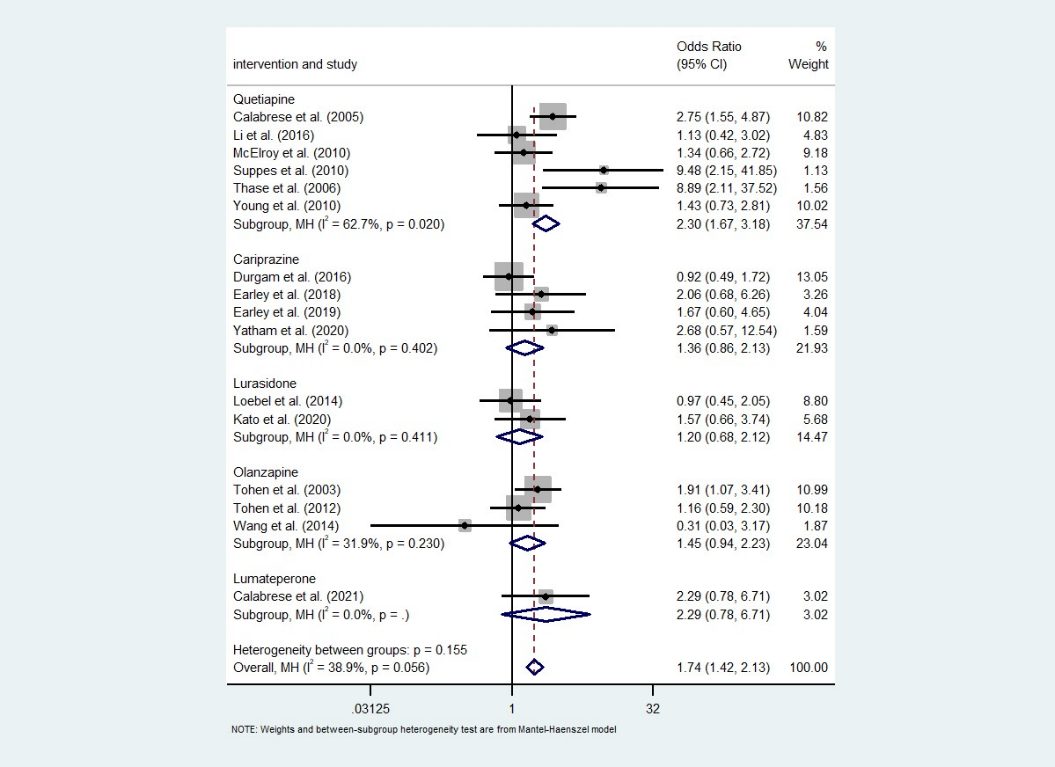**eFig. 1d.** Forest plot for the discontinuation due to the adverse events using a fixed effects model. The Q test and *I^2^* statistic were used to test the heterogeneity. CI, confidence interval. |
| --- | --- |
| 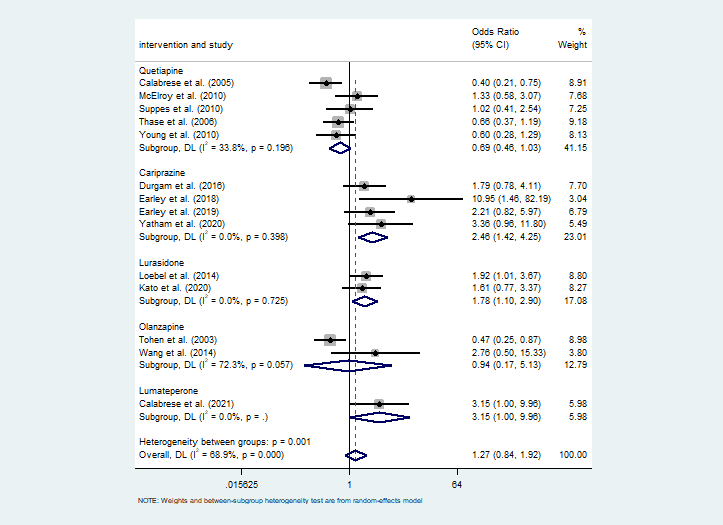**eFig. 2a.** Forest plot for the rate of nausea using a random effects model. The Q test and *I^2^* statistic were used to test the heterogeneity. CI, confidence interval. | 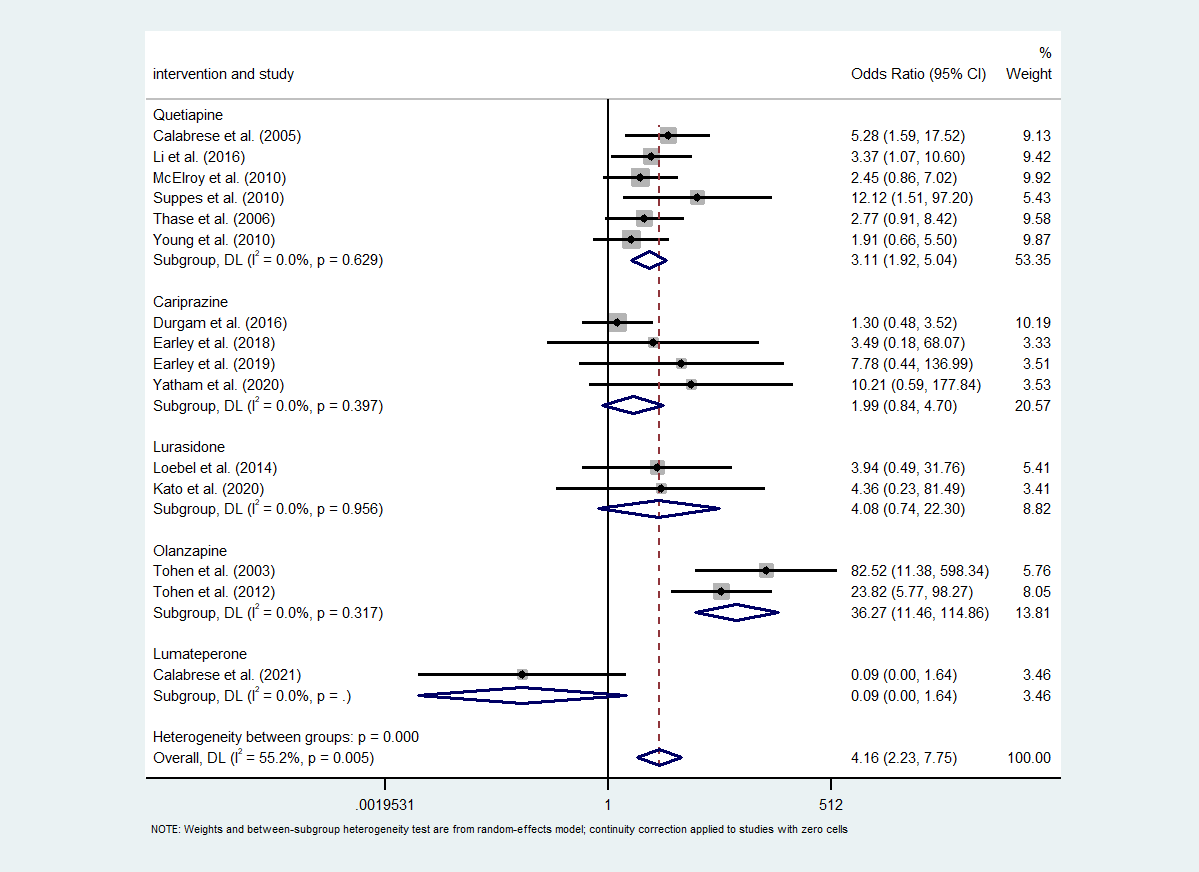**eFig. 2b.** Forest plot for the rate of ≥7% weight gain after the treatment using a random effects model. The Q test and *I^2^* statistic were used to test the heterogeneity. CI, confidence interval. |
| 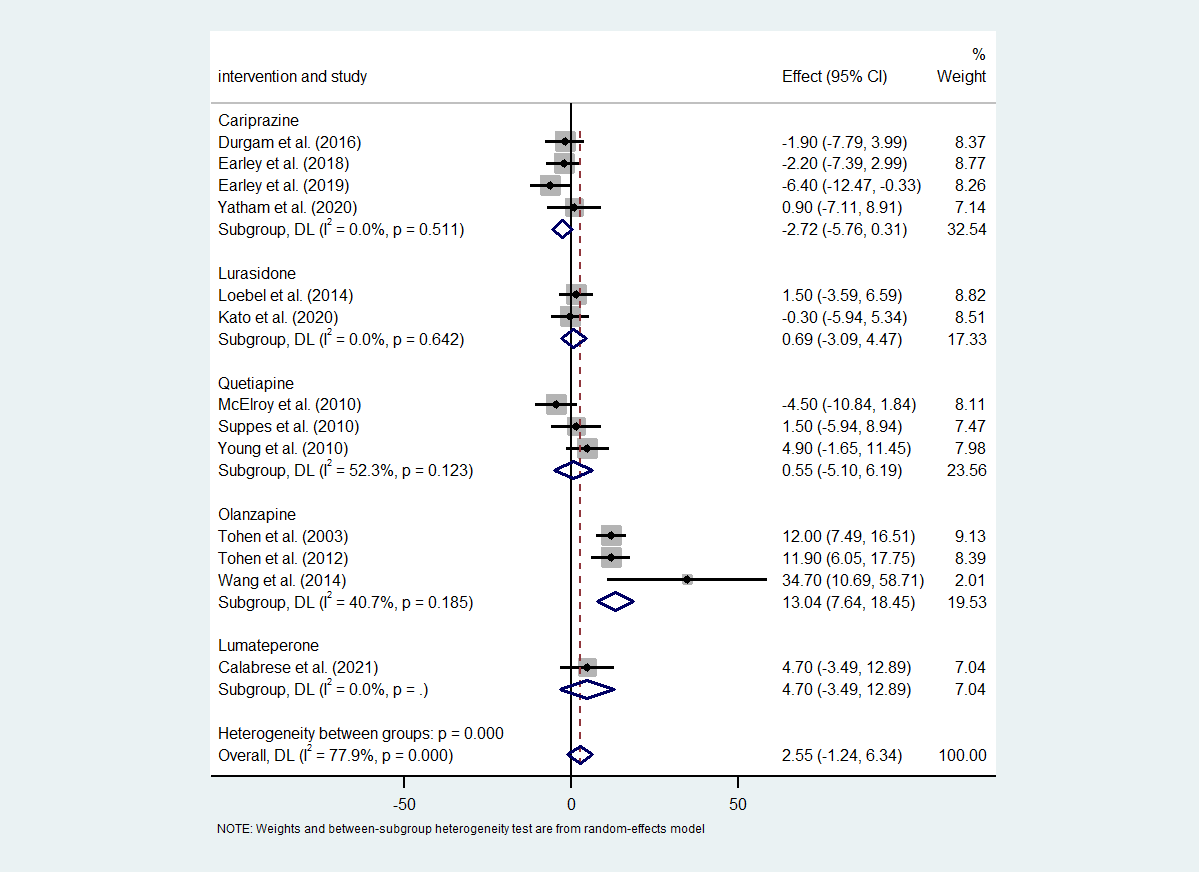**eFig. 2c.** Forest for the change from baseline in total cholesterol after the treatment using a random effects model. The Q test and *I^2^* statistic were used to test the heterogeneity. CI, confidence interval.  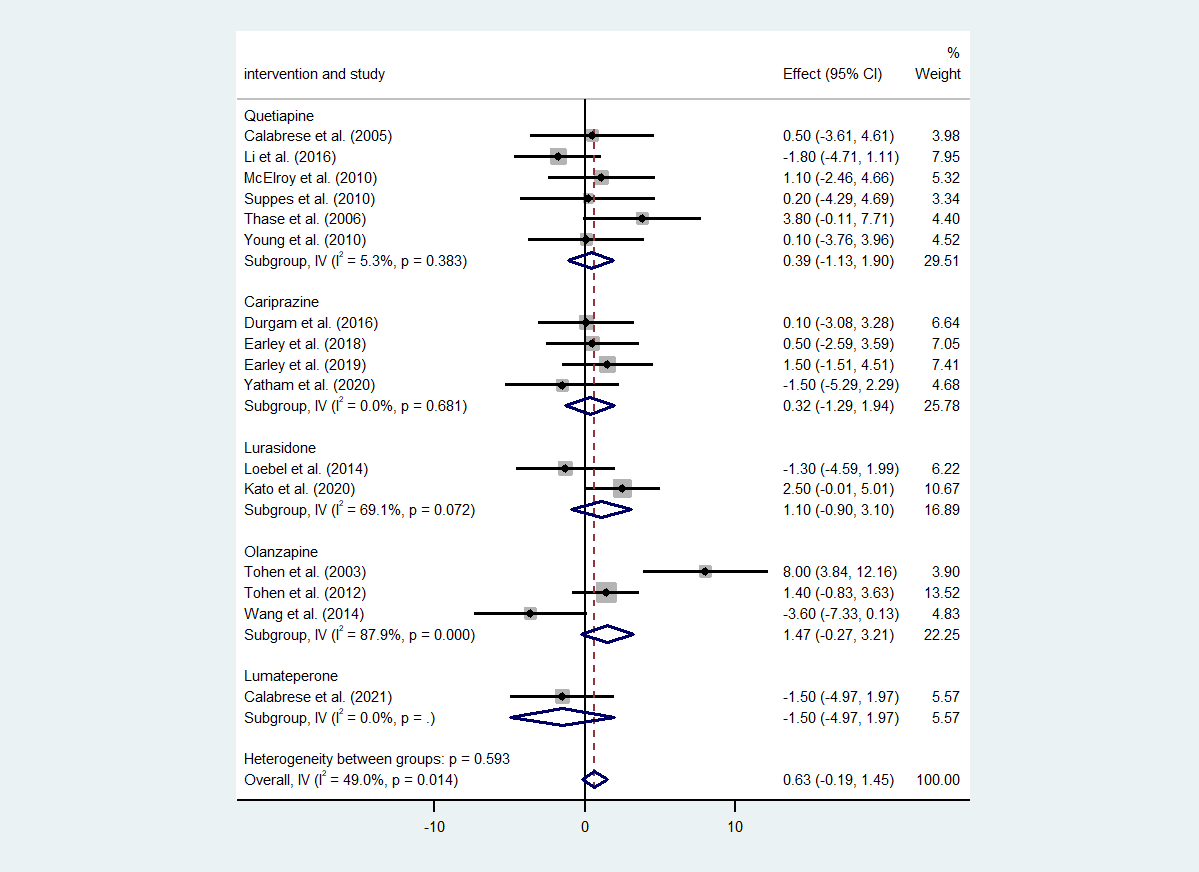**eFig. 2e.** Forest for the change from baseline in blood glucose after the treatment using a fixed effects model. The Q test and *I^2^* statistic were used to test the heterogeneity. CI, confidence interval. | **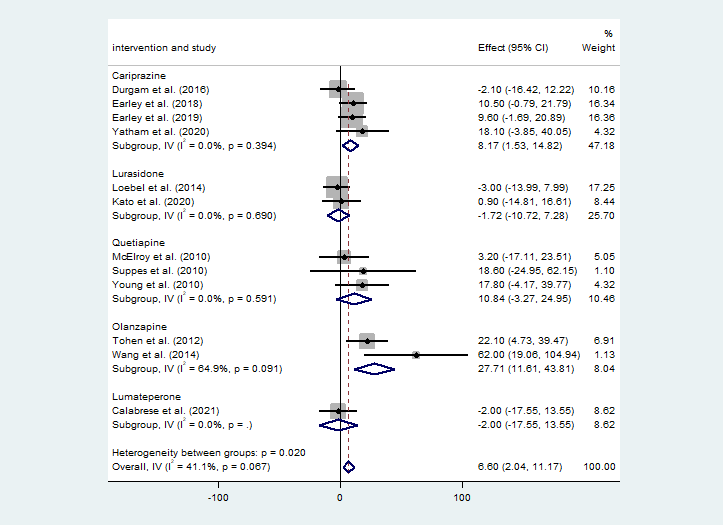eFig. 2d.** Forest for the change from baseline in triglycerides after the treatment using a fixed effects model. The Q test and *I^2^* statistic were used to test the heterogeneity. CI, confidence interval. |


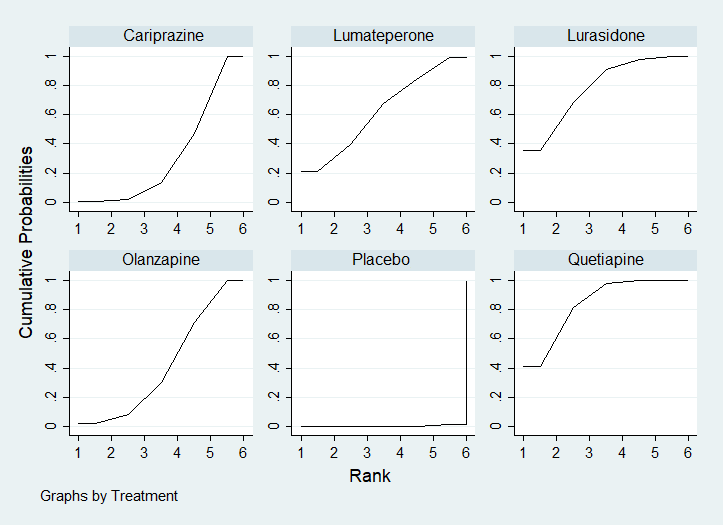


**eFig. 3.** Cumulative probability ranking plot of response rate (


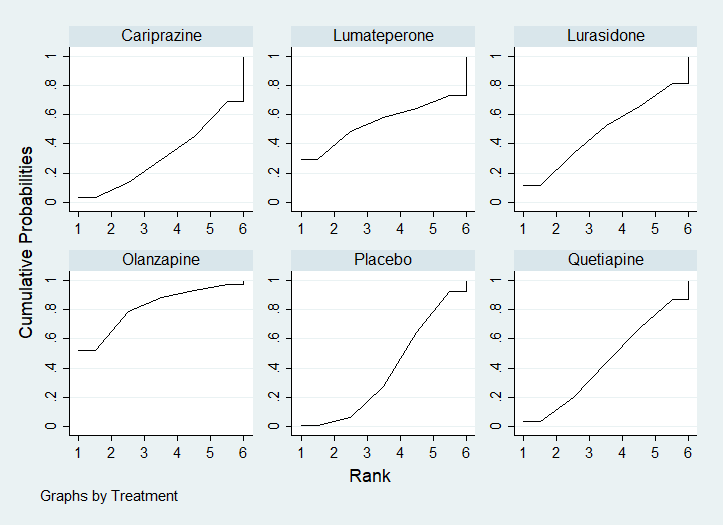


**eFig. 4.** Cumulative probability ranking plot of all-cause discontinuation (SUCRA).


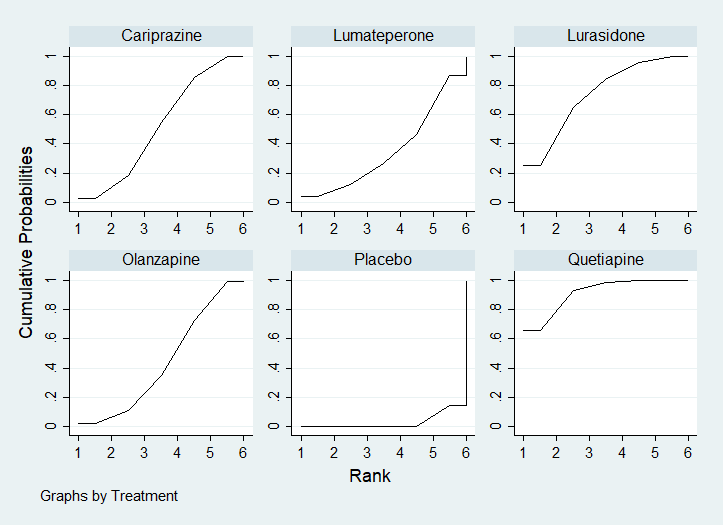


**eFig. 5.** Cumulative probability ranking plot of remission rate (SUCRA).


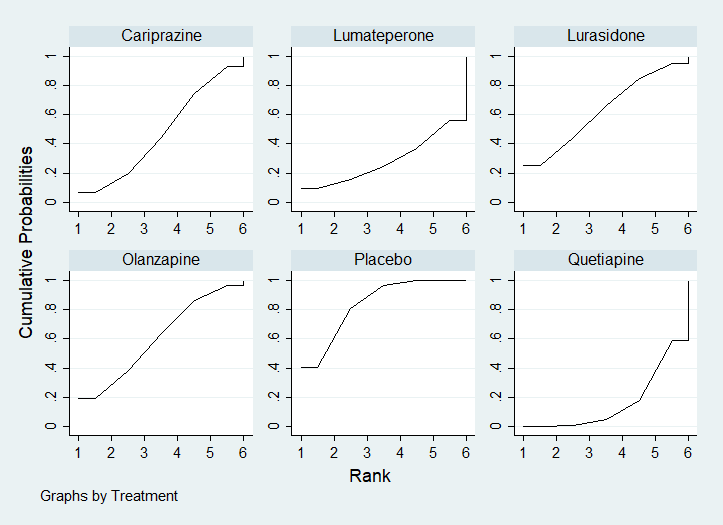


**eFig. 6.** Cumulative probability ranking plot of discontinuation due to adverse events (SUCRA).


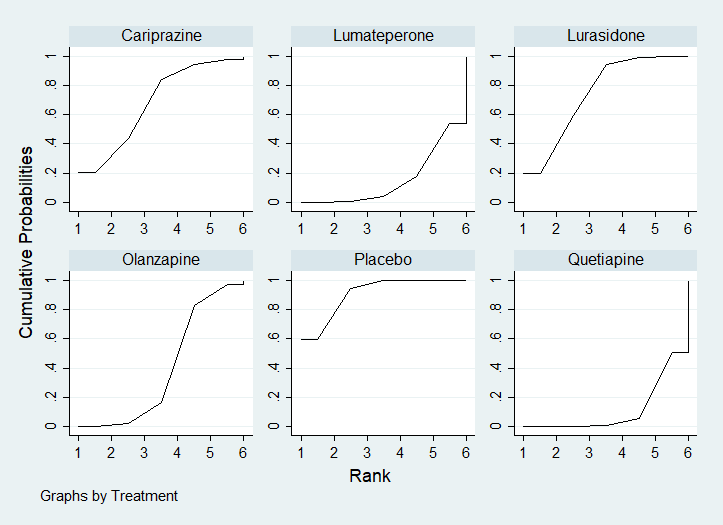


**eFig. 7.** Cumulative probability ranking plot of somnolence rate (SUCRA).


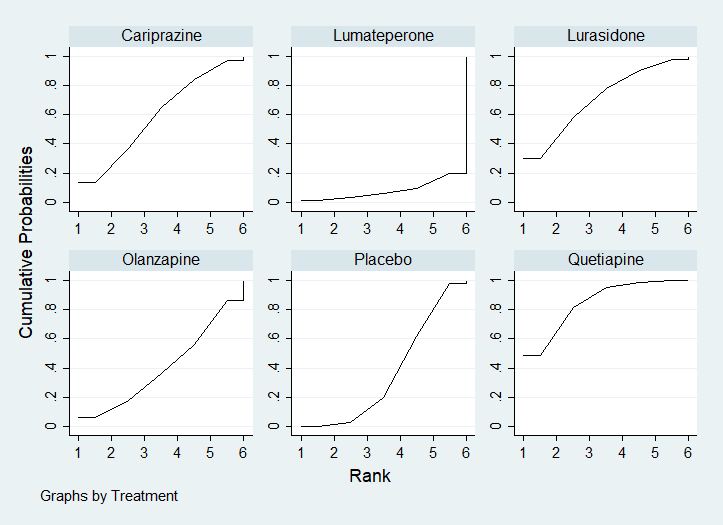


**eFig. 8.** Cumulative probability ranking plot of headache rate (SUCRA).


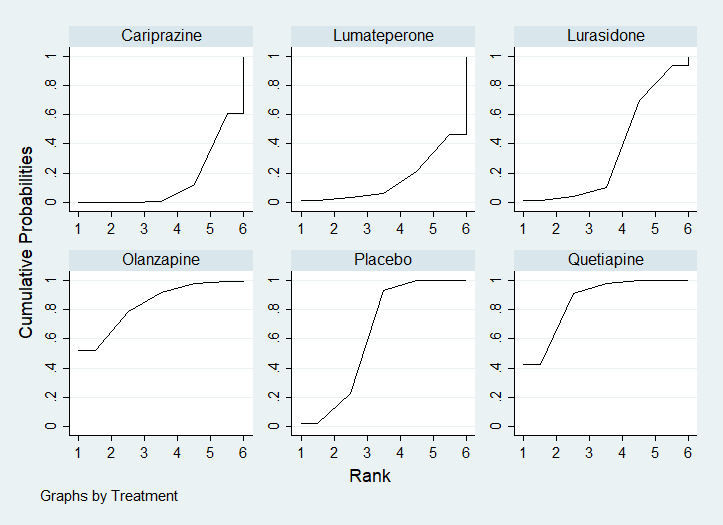


**eFig. 9.** Cumulative probability ranking plot of nausea rate (SUCRA).


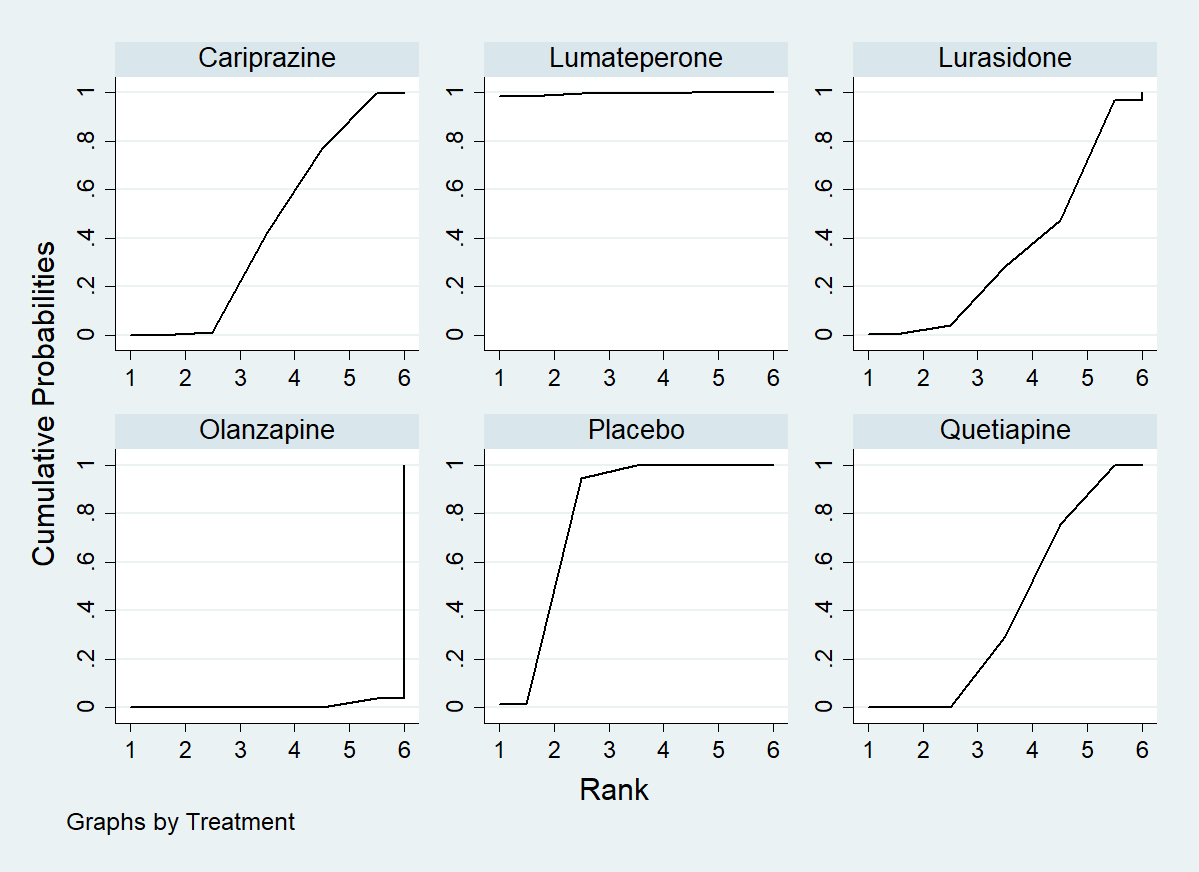


**eFig. 10.** Cumulative probability ranking plot of ≥7% weight gain rate (SUCRA).


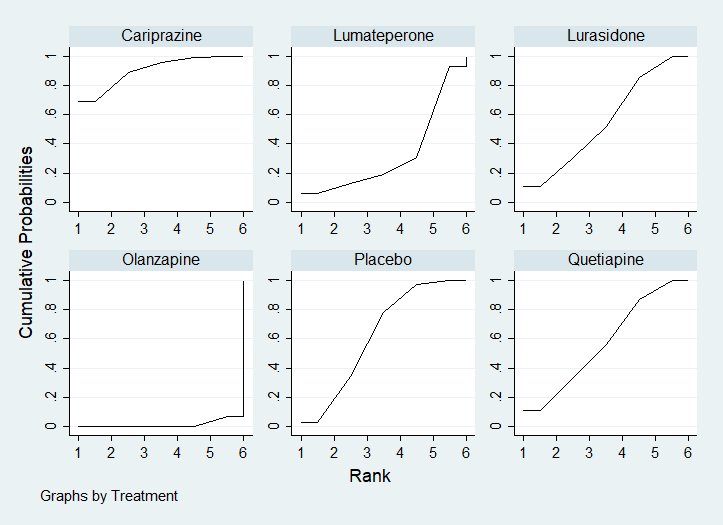


**eFig. 11.** Cumulative probability ranking plot of change from baseline in total cholesterol (SUCRA).


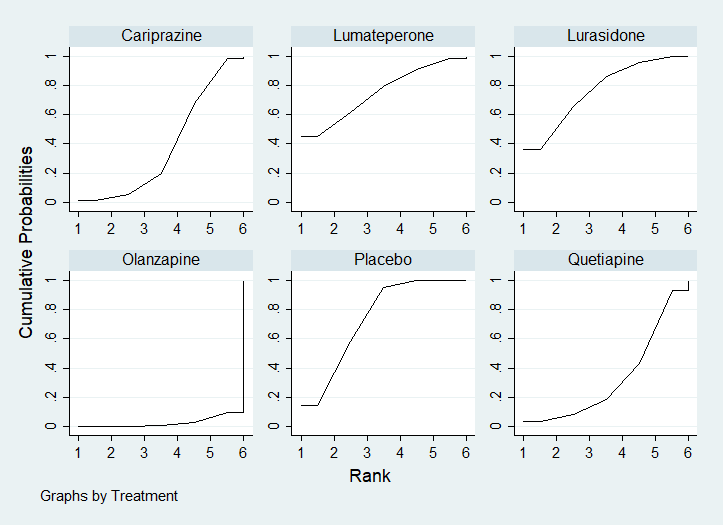


**eFig. 12.** Cumulative probability ranking plot of change from baseline in triglycerides


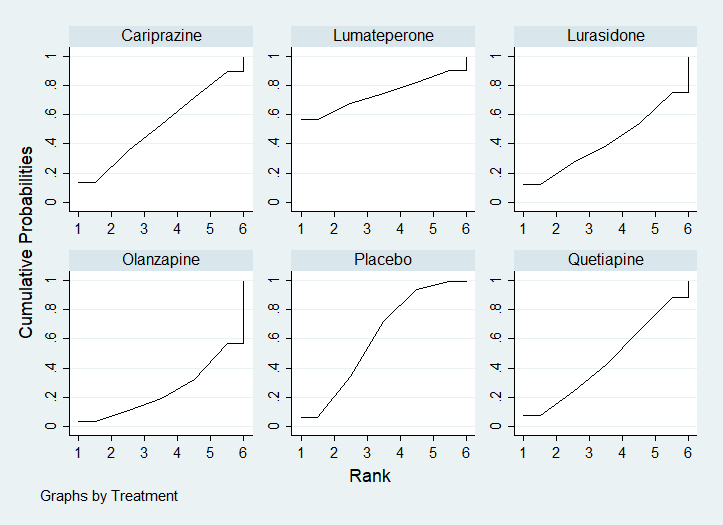


**eFig. 13.** Cumulative probability ranking plot of change from baseline in blood glucose (SUCRA).


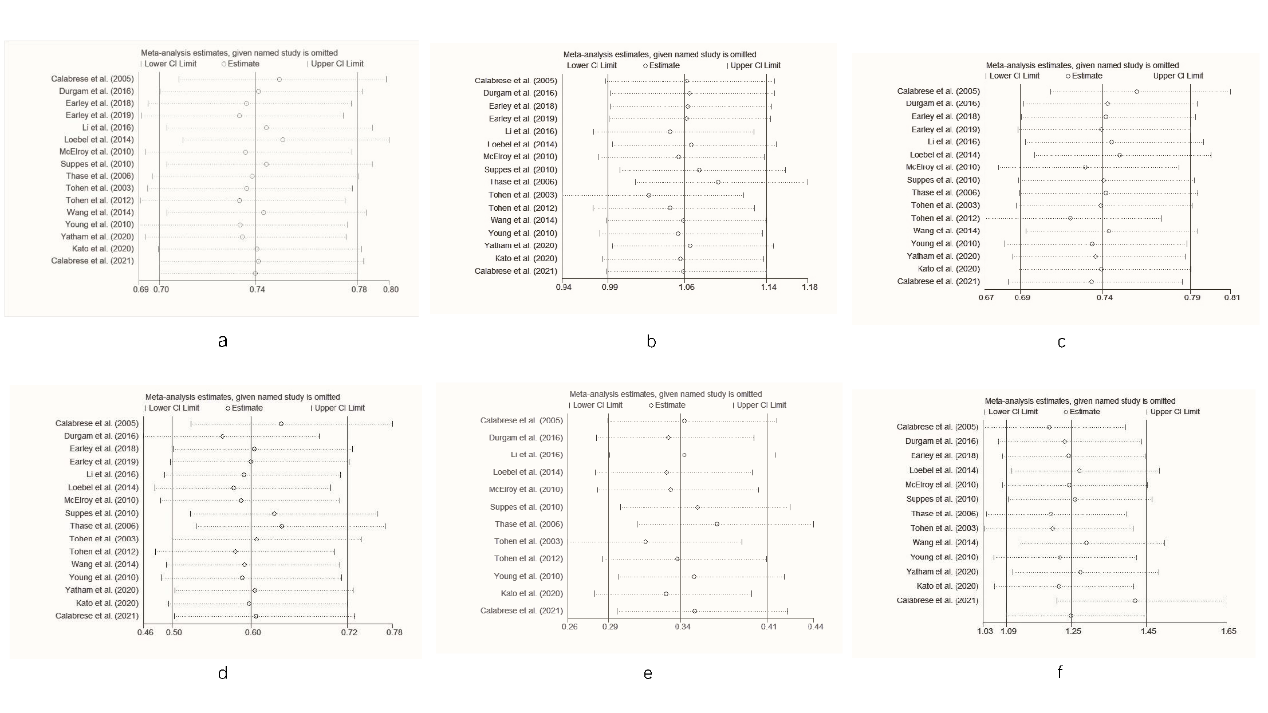


**eFig. 14.** (a) Sensitivity analysis for the response rate (b) Sensitivity analysis for the all-cause discontinuation (c) Sensitivity analysis for the remission rate (d) Sensitivity analysis for the discontinuation due to the adverse events (e) Sensitivity analysis for the rate of somnolence (f) Sensitivity analysis for the rate of headache


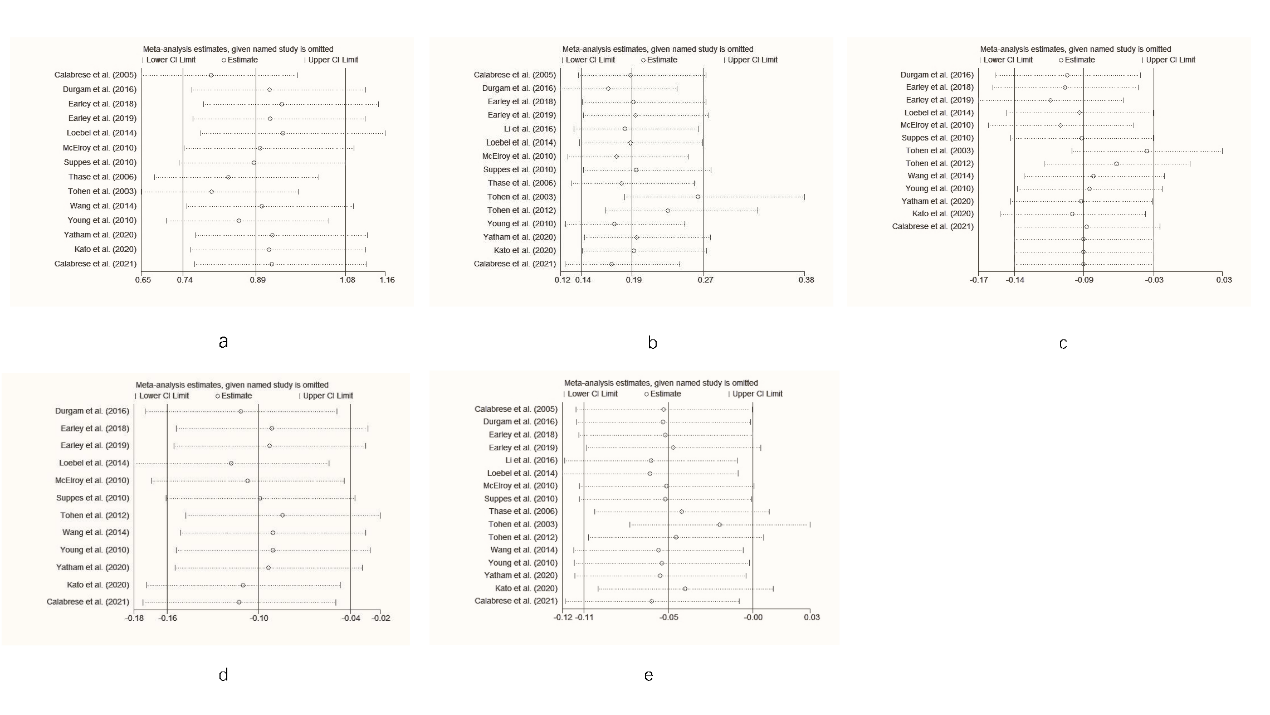


**eFig. 15.** (a) Sensitivity analysis for the rate of nausea (b) Sensitivity analysis for the rate of ≥7% weight gain (c) Sensitivity analysis for the change from baseline in total cholesterol (d) Sensitivity analysis for the change from baseline in triglycerides (e) Sensitivity analysis for the change from baseline in blood glucose


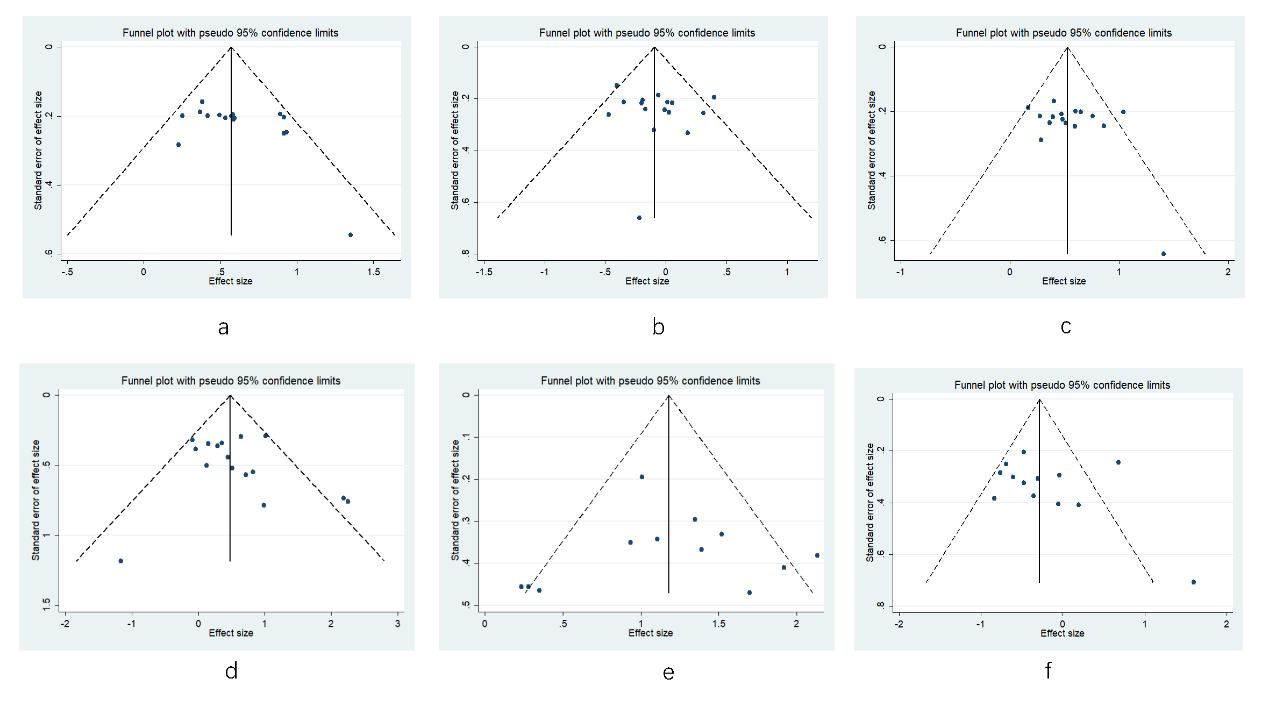


**eFig. 16.** (a) Funnel plot for the response rate (b) Funnel plot for the all-cause discontinuation (c) Funnel plot for the remission rate (d) Funnel plot for the discontinuation due to the adverse events

(e) Funnel plot for the rate of somnolence (f) Funnel plot for the rate of headache


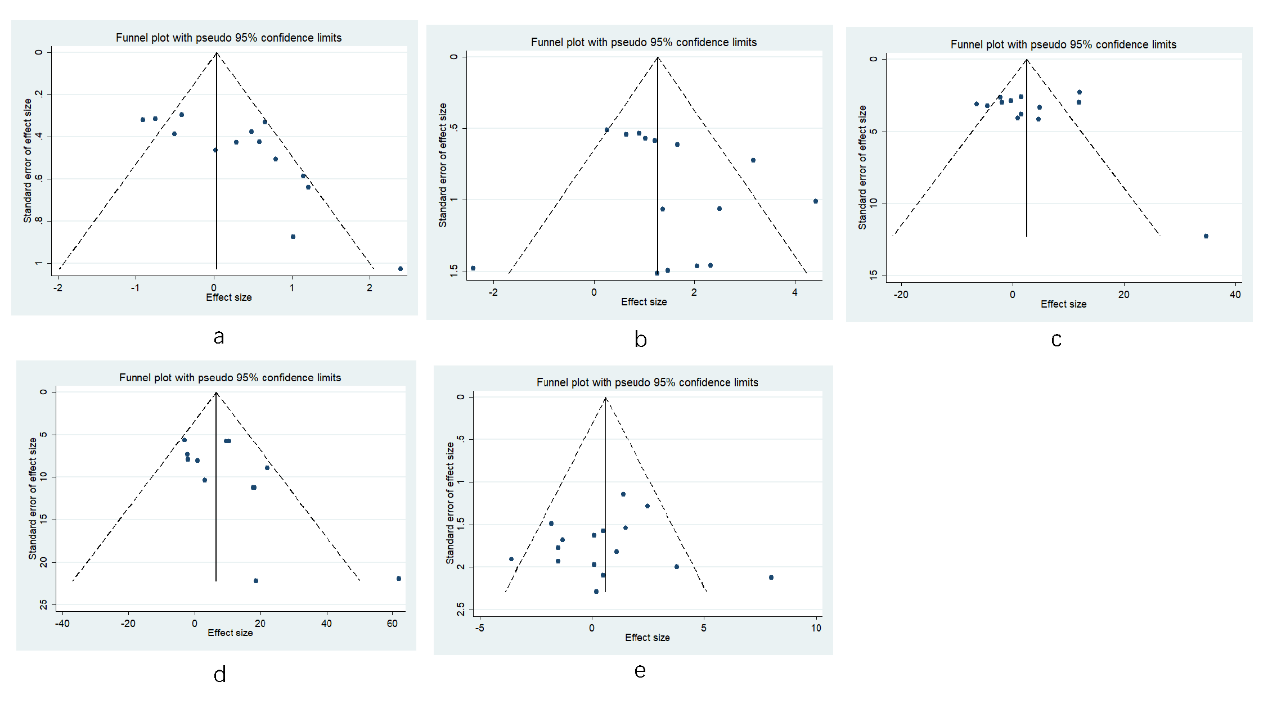


**eFig. 17.** (a) Funnel plot for the rate of nausea (b) Funnel plot for the rate of ≥7% weight gain (c) Funnel plot for the change from baseline in total cholesterol (d) Funnel plot for the change from baseline in triglycerides (e) Funnel plot for the change from baseline in blood glucose


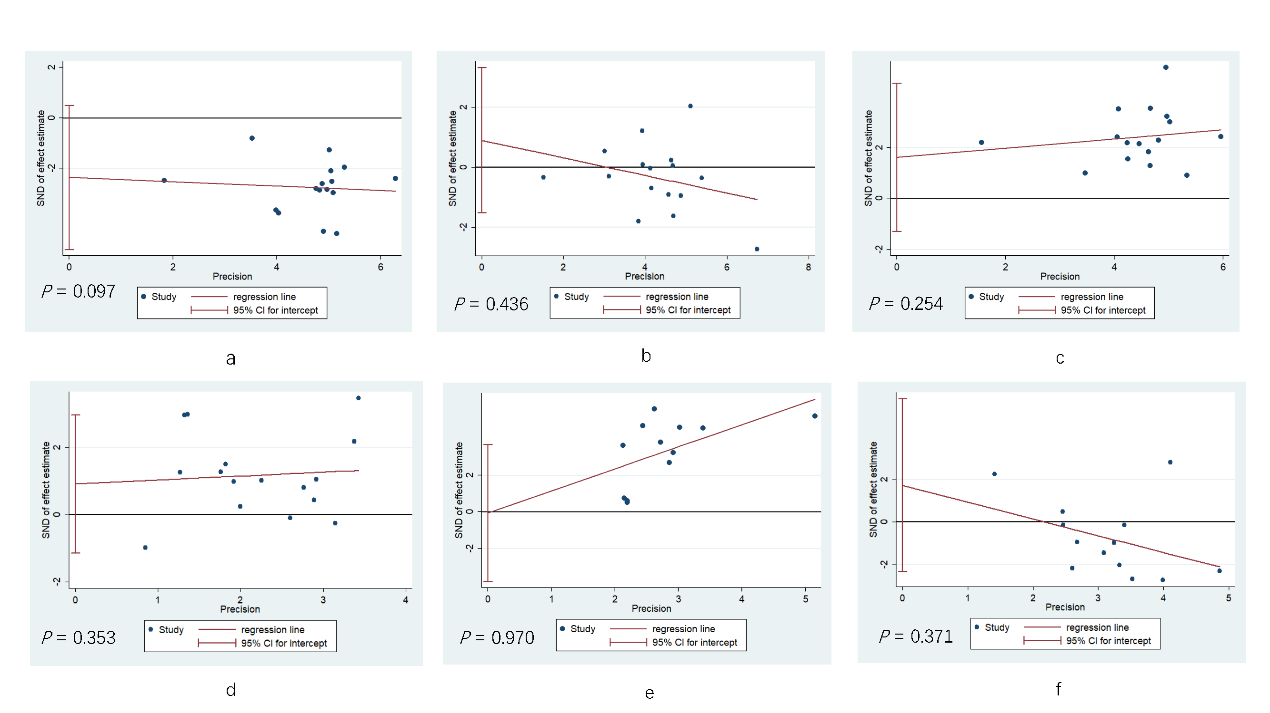


**eFig. 18.** (a) Egger’s test for the response rate (b) Egger’s test for the all-cause discontinuation (c) Egger’s test for the remission rate (d) Egger’s test for the discontinuation due to the adverse events

(e) Egger’s test for the rate of somnolence (f) Egger’s test for the rate of headache


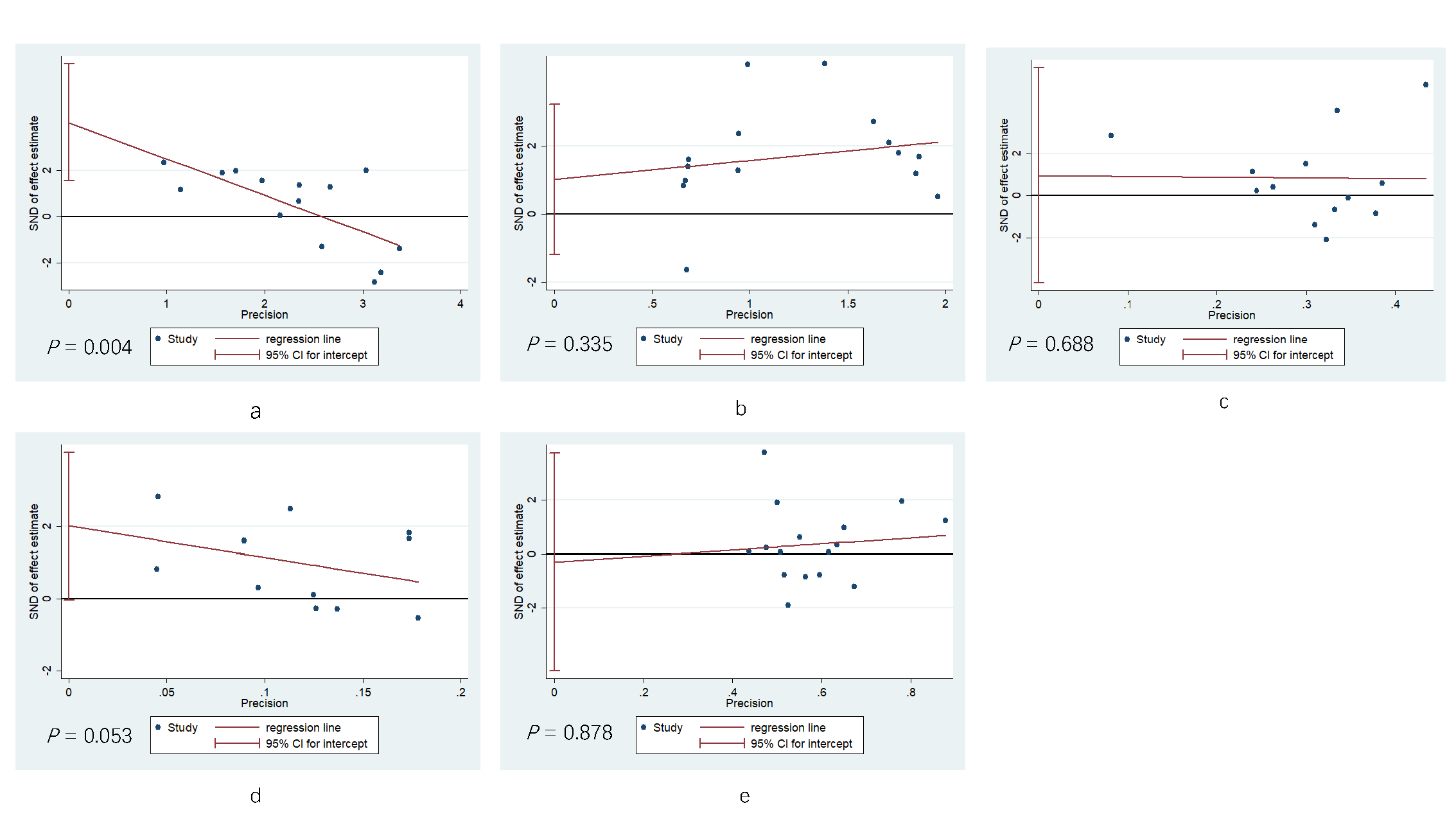


**eFig. 19.** (a) Egger’s test for the rate of nausea (b) Egger’s test for the rate of ≥7% weight gain (c) Egger’s test for the change from baseline in total cholesterol (d) Egger’s test for the change from baseline in triglycerides (e) Egger’s test for the change from baseline in blood glucose
